# Longitudinal Changes in Epigenetic Age Acceleration Across Childhood and Adolescence

**DOI:** 10.1001/jamapediatrics.2024.3669

**Published:** 2024-10-07

**Authors:** Juan Del Toro, Connor Martz, Colin D. Freilich, Gianna Rea-Sandin, Kristian Markon, Steve Cole, Robert F. Krueger, Sylia Wilson

**Affiliations:** 1 Department of Psychology, University of Minnesota-Twin Cities, Minneapolis; 2 Population Research Center, University of Texas-Austin, Austin; 3 Department of Pediatrics, University of Minnesota-Twin Cities, Minneapolis; 4 School of Medicine, University of California, Los Angeles; 5 Institute of Child Development, University of Minnesota-Twin Cities, Minneapolis

## Abstract

**Importance:**

Individuals exposed to discrimination may exhibit greater epigenetic age acceleration (ie, cellular indicators of premature aging) over time, but few studies have examined longitudinal changes in epigenetic age acceleration, the heterogeneity in these changes for diverse groups of youths, and contextual explanations (ie, discrimination) for differences by ethnicity or race.

**Objective:**

To provide a descriptive illustration of changes in epigenetic age acceleration across childhood and adolescence among an ethnically and racially diverse sample of youths.

**Design, Setting, and Participants:**

This cohort study leveraged longitudinal data on a large sample of youths from low-income households in 20 large urban US cities who provided repeated assessments of saliva tissue samples at ages 9 and 15 years for DNA methylation analysis. Of 4898 youths from the Future of Families and Child Well-Being study, an ongoing study that oversampled children born to unmarried parents from 1998 to 2000, 2039 were included in the present analysis, as these youths had salivary DNA methylation data assayed and publicly available. Analyses were conducted from March 2023 to June 2024.

**Exposures:**

Racialized intrusive encounters with police (eg, stop and frisk and racial slurs).

**Main Outcomes and Measures:**

Analyses were conducted to examine longitudinal changes in salivary epigenetic age acceleration over time, whether such changes varied across ethnically and racially diverse groups of youths, and whether police intrusion was associated with variation across ethnic and racial groups.

**Results:**

Among 2039 youths (mean [SD] age at baseline, 9.27 [0.38] years; 1023 [50%] male and 1016 [50%] female; 917 [45%] Black, 430 [21%] Hispanic or Latino, 351 [17%] White, and 341 [17%] other, including multiple races and self-identified other) with salivary epigenetic clocks at 9 and 15 years of age, longitudinal results showed that White youths exhibited less accelerated epigenetic aging over time than did Black and Hispanic or Latino youths and those reporting other or multiple races or ethnicities from ages 9 to 15 years, particularly in the Hannum (B, 1.54; 95% CI, 0.36-2.18), GrimAge (B, 1.31; 95% CI, 0.68-1.97), and DunedinPACE epigenetic clocks (B, 0.27; 95% CI, 0.11-0.44). Across these clocks and the PhenoAge clock, police intrusion was associated with Black youths’ more accelerated epigenetic aging (Hannum: B, 0.11; 95% CI, 0.03-0.23; GrimAge: B, 0.09; 95% CI, 0.03-0.18; PhenoAge: B, 0.08; 95% CI, 0.02-0.18; DunedinPACE: B, 0.01; 95% CI, 0.01-0.03).

**Conclusions and Relevance:**

The transition from childhood to adolescence may represent a sensitive developmental period when racism can have long-term deleterious impacts on healthy human development across the life span. Future research should build on the present study and interrogate which social regularities and policies may be perpetuating discrimination against ethnically and racially minoritized adolescents.

## Key Points

**Question:**

Does epigenetic age acceleration change as youths transition from childhood to adolescence, do these changes in epigenetic age acceleration differ between youths from diverse ethnic and racial backgrounds, and do interpersonal manifestations of discrimination explain these ethnic and racial group differences?

**Findings:**

In this cohort study of 2039 US youths, those who were White exhibited less accelerated epigenetic aging over time than did youths who were Black, Hispanic or Latino, or multiple or self-identified other race or ethnicity from ages 9 to 15 years, particularly in the Hannum, GrimAge, and DunedinPACE clocks. For Black youths, these differences in epigenetic age acceleration were partly attributable to exposures to police intrusion (eg, stop and frisks and racial slurs).

**Meaning:**

The findings suggest that addressing interpersonal and structural discrimination in policing may benefit the health of youths of minoritized racial and ethnic groups in the US.

## Teaser

This cohort study evaluates differences in epigenetic age acceleration by race and ethnicity.

## Introduction

Discrimination is a fundamental cause of ethnic and racial inequities in healthy human aging.^1^ Discrimination operates via cultural processes, policies, and institutions that benefit White US individuals to the detriment of US individuals of ethnically and racially minoritized groups.^2^ Ethnic and racial discrimination can undermine individuals’ self-esteem, lead to rumination, and prime vigilance to more discrimination.^3,4^ In addition, redlining and segregation have pigeonholed underrepresented groups to live in communities affected by concentrated poverty, urban disinvestment, and pervasive police surveillance and harassment.^5,6,7^ On average, historically ethnically and racially marginalized groups in the US have exhibited faster paces of biological aging^8,9^ and shorter life spans than White individuals in the US.^10^

One theorized mechanism for the biological embedding of discrimination is DNA methylation.^11,12^ Recent technological advances have led to the assessment of accelerated biological aging via epigenetic clocks, which are composite measures of DNA methylation sites that correlate with aging-related phenotypes (ie, chronological age, illness, and mortality).^13,14^ These epigenetic clocks include first-generation measures, trained to estimate a person’s chronological age, and second- and third-generation measures, which were trained to predict mortality risk and pace of change in aging-related biomarkers.^13,14^ Given ethnic and racial disparities in phenotypic aging,^8,10^ it is plausible to expect ethnic and racial differences in epigenetic age acceleration (EAA; ie, the difference between epigenetic age and chronological age).

Several existing conceptual frameworks highlight how discrimination can have negative health outcomes. Ecosocial theory highlights the historical and structural dynamics driving health disparities.^15^ Minority stress theory posits distal (eg, biased policies) and proximal stressors (eg, discrimination) that lead to health inequities.^16^ The weathering hypothesis emphasizes the chronicity of exposures to social and economic disadvantages that accelerate decline in health among communities of minoritized racial or ethnic groups.^17^ Altogether, these frameworks showcase the multiple pathways and levels of society through which discrimination can become biologically embodied.^15^

Although theories highlight disparities in exposures to disadvantage between ethnic and racial groups, these disparities are not entirely reflected in epigenetic aging. In a sample of 4535 participants, Black US individuals showed slower EAA than White US individuals in a first-generation clock (ie, Horvath).^18^ Similarly, in one of the largest EAA studies of retiring adults (ie, the Health and Retirement Study^19,20,21^), Black and Hispanic or Latino US individuals exhibited lower EAA in first-generation clocks (eg, Hannum) but faster EAA in most second-generation clocks (eg, DunedinPoAm) than their White peers. These counter-hypothetical findings are partly attributable to recent advances in epigenetic clocks to predict health measures in second- and third-generation clocks as opposed to primarily chronological aging in first-generation clocks. Indeed, in recent studies^22,23^ of youths ages 8 to 18 years, Black and Hispanic or Latino US individuals showed greater EAA in the DunedinPoAm clock but not in the other clocks. Because most pediatric epigenetic studies come from homogenous and small community samples,^24^ more research is needed to understand the possible role of EAA in the biological embedding of structural discrimination, especially among youths of ethnically and racially minoritized groups who are underrepresented in epigenetic research.

Interpersonal manifestations of discrimination, such as self-reported discrimination, may explain differences in epigenetic aging between ethnic and racial groups, but empirical results have not provided robust results in line with extant theories. In a large diverse study of midlife adults (ie, the Midlife in the United States [MIDUS] study^25^), discrimination was unrelated to all 3 generations of epigenetic clocks. Surprisingly, across the second-generation epigenetic clocks, discrimination was unrelated to EAA among Black adults in the MIDUS study,^26^ the Health and Retirement Study,^27^ and a low-income community sample.^28^ One potential explanation for extant studies’ pattern of findings is their shared focus on adults, who have more developed coping strategies in response to discrimination than children and adolescents.^29^

The developmental timing of exposures can have implications for change in EAA, according to life course theory.^14,30^ A life course perspective suggests that cumulative exposures occurring during sensitive developmental periods can have additive effects on the epigenome, thus increasing individuals’ biological susceptibilities to EAA.^14,30^ Thus, changes in EAA over time are possible and may be uniquely sensitive during developmental transitions, such as that between childhood and adolescence. However, given most epigenetic studies are cross-sectional,^31^ longitudinal changes in EAA and their sensitivity to discrimination during developmental transitions have not been empirically tested.

The current study aimed to assess potential sources of ethnic and racial differences in EAA across late childhood and midadolescence, using longitudinal salivary EAA data when youths were ages 9 and 15 as part of the Future of Families and Child Well-Being (FFCW) study. The FFCW study is an ongoing national study of youths primarily born from nonmarital births between 1998 and 2000, which reflected about one-third of youths at the time and 40% of births in 2016.^32^ Thus, the data from the FFCW study carries external validity in being representative of a considerable portion of the US population and who are typically underrepresented in pediatric national samples. These samples, including indvididuals from the Avon Longitudinal Study of Parents and Children (ALSPAC), the Texas Twin Project, and the E-Risk Study, have illustrated that epigenetic clocks trained on adults perform well in children and adolescents.^31^ In line with extant theories, we predicted that ethnic and racial differences in EAA would appear early in the life course. In addition, we hypothesized that racialized police intrusive encounters (eg, stop and frisk and racial slurs), as sources of discrimination,^7,33^ would explain associations between youths’ ethnic and racial backgrounds and EAA.

## Methods

### Participants

The FFCW study included 4898 youths, of whom 2039 had DNA methylation data and were included in this study.^34^ The eAppendix in Supplement 1 includes the study procedure and exclusionary criteria from the initial sample of 4898. Included families were mostly low-income; 321 mothers [82%] did not have a 4-year college or more advanced degree, and most mothers were initially recruited as part of the study as single mothers. Race and ethnicity were assessed via parent or guardian self-report using the following categories defined by study protocol: American Indian, Asian or Pacific Islander, Black or African-American, Eskimo Eleut, Hispanic, White, and other. Groups with small numbers were combined to protect participant privacy. Table 1 presents demographic information. We followed the Strengthening the Reporting of Observational Studies in Epidemiology (STROBE) reporting guideline. The institutional review boards at the University of Michigan and Princeton University granted ethical approval. Informed written consent was obtained from youths’ legal guardians and assent from participating youths.

**Table 1.** Participants’ Demographic Characteristics

Measure	Race and ethnicity, mean (SD)				ANOVA or χ^2^	*P* value
	Black (n = 917)	Hispanic/Latino (n = 430)	White (n = 351)	Other (n = 341)^a^		
Sex, No. (%)						
Female	460 (50.20)	217 (50.50)	170 (48.40)	169 (49.60)	χ^2^_3_ = 0.39	.94
Male	457 (49.80)	213 (49.50)	181 (51.60)	172 (50.40)		
Youth age at baseline, y	9.24 (0.39)	9.36 (0.38)	9.19 (0.30)	9.27 (0.43)	*F*_3,2026_ = 13.59	<.001
Maternal education	2.49 (0.89)	2.05 (0.99)	3.04 (0.98)	2.69 (1.02)	*F*_3,1978_ = 72.34	<.001
Youth smoking, No. (%)	8 (0.90)	3 (0.70)	5 (1.40)	3 (0.90)	χ^2^_3_ = 1.26	.74
Maternal smoking, No. (%)	183 (20.00)	32 (7.50)	102 (29.10)	71 (20.80)	χ^2^_3_ = 61.68	<.001
Youth BMI	1.00 (0.95)	1.05 (0.92)	0.64 (0.91)	0.88 (0.97)	*F*_3,1873_ = 13.81	<.001
Youth pubertal development	0.96 (0.57)	0.95 (0.53)	0.99 (0.47)	0.94 (0.55)	*F*_3,2024_ = 0.62	.60
Police intrusion	0.47 (1.15)	0.25 (0.81)	0.12 (0.54)	0.21 (0.71)	*F*_3,2015_ = 15.44	<.001

Abbreviations: ANOVA, analysis of variance; BMI, body mass index (calculated as weight in kilograms divided by height in meters squared).

^a^ Other race and ethnicity groups included multiple races and self-identified other.

### Measures

#### EAA

The FFCW study constructed DNA methylation measures that estimated epigenetic age from saliva samples collected from youths at ages 9 and 15 years. Saliva samples were assayed for genome-wide DNA methylation profiling using Illumina EPIC or 450K methylation arrays for 874 and 1165 participants, respectively. In addition to standard quality control protocols at each wave, a participant’s age 9- and 15-year samples were run at the same time to minimize technical variation. Additional details about data collection and processing are available online via the FFCW study portal.^35^ The 2039 profiles at each wave were scored using published algorithms to compute several measures of epigenetic age, including the Horvath,^36^ Hannum,^37^ PhenoAge,^38^ GrimAge,^39^ and DunedinPACE clocks.^40^ Consistent with extant recommendations,^41^ array data were pooled and detrended to remove differences between arrays, as multigroup χ^2^ tests suggested that our main findings did not differ between the 2 Illumina arrays for the Horvath (Δχ^2^_6_ = 5.11; *P* = .53), Hannum (Δχ^2^_6_ = 10.09; *P* = .12), PhenoAge (Δχ^2^_6_ = 8.99; *P* = .17), GrimAge (Δχ^2^_6_ = 10.13; *P* < .12), and DunedinPACE (Δχ^2^_6_ = 5.29; *P* < .51) clocks. The clocks were also regressed on chronological age to obtain residual scores for EAA, except for the DunedinPACE clock, which was standardized. Values greater than 0 indicate greater EAA; values lower than 0 are indicative of less EAA.

Because most epigenetic clocks were derived from homogenous samples of adults, we examined whether such clocks equally correlated with metrics of aging and health to assess criterion-related (ie, concurrent, parallel, and predictive) validity of our measures among our heterogenous sample of youths. Evidence of criterion-related validity of our clocks emerged among the present ethnically and racially diverse sample of youths (eAppendix in Supplement 1).

#### Ethnicity and Race

Youths’ ethnicity and race was coded based on their biological mothers’ and fathers’ self-reported ethnicity and race. These parental reports were significantly correlated with adolescents’ self-identification at age 15 years.

#### Police Intrusion

At age 15 years, adolescents responded to the 6-item Police Intrusion Scale^42^ and reported their most memorable police intrusive encounter (“Did the officer frisk or use racial slurs?” 0 = no, 1 = yes; α, 0.76), which occurred as early as age 8 years and at a mean (SD) age of 13.29 (1.71) years. High scores reflected more types of intrusion that youths directly and most memorably experienced.

#### Covariates and Missing Data

Table 2^43,44,45,46,47^ includes a list of covariates. Among the 2039 youths and constructs listed in Table 2, 1691 youths (83%) had complete data on all measures; 211 (10%) had 1 variable missing; 4 (0.20%) had 2 variables missing; 115 (6%) had 6 variables missing; 10 (0.50%) had 7 variables missing; 3 (0.10%) had 9 variables missing; and 5 (0.20%) had 11 variables missing. The count of missing variables was unrelated to youths’ race, sex, age, maternal education, and body mass index. After adjusting for these covariates, semipartial correlations showed that the count of missing data was unrelated to each EAA outcome (eAppendix in Supplement 1). According to Enders,^48^ because our missing data were unrelated to our key measures (ie, EAA), our data were conditionally missing at random. Results from our main analysis were similar to findings that emerged among the 83% subset with complete data available. To retain sample variability and diversity, missing data were handled by the full-information Markov Chain Monte Carlo method in bayesian estimation.^49^

**Table 2.** List of Covariates

Variable	Description
Youth sex	Measured on a 2-point Likert scale (0 = female, 1 = male).
Youth age	Mean (range), 9 (8.67-11.92) y. When examining EAA, Krieger et al^43^ recommend either analyzing accelerated aging detrended for age as the outcome and control for age as a covariate (ie, our main analysis) or analyzing raw DNA methylation age as the outcome and control for age as a covariate, which produced similar results as our main analysis (eTables 5-6 in Supplement 1).
Family socioeconomic status at birth	At birth, an average score was computed using standardized parental educational attainment and standardized log-transformed parental income. This score was more predictive of DNA methylation than family socioeconomic status at ages 9 or 15 y.^44^ In zero-order bivariate correlations, neither maternal or paternal education at birth (1 = less than high school degree, 2 = high degree or equivalent, 3 = some college or technical experience, 4 = college or advanced degree) showed evidence of nonlinear associations with any of the EAA clocks (eTable 2 in Supplement 1).
Youth smoking	At age 9 y, children completed a 1-item assessment (“Have you ever smoked a cigarette or used tobacco?”) measured with a 2-point Likert scale (0 = no, 1 = yes).
Maternal smoking	At pregnancy, mothers completed a 1-item assessment (“During the pregnancy, how many cigarettes did you smoke?”; measured as 0 = none, 1 = less than 1 pack a day, 2 = between 1 and 2 packs a day, 3 = 2 or more packs a day), which was transformed to reflect whether mothers smoked or not during pregnancy (0 = no, 1 = yes).
DNA methylation score for smoking	At ages 9 and 15 y, DNA methylation smoking (packs/year) was computed on the basis of an epigenome-wide association study of adult smoking and subsequently developed for youths.^44^ This score was residualized for Illumina array, batch, and saliva-based cell composition and standardized.
BMI	At age 9 y, children’s BMI was standardized (range, −4.16 to 4.74).
Youth pubertal development	At age 9 y, parents reported their children’s pubertal development (1 item: “Compared with other [boys/girls] [his/her] age, do you think [child]’s physical development is…”), on a 3-point Likert scale (0 = earlier, 1 = about the same, 2 = later).
Illumina array	At ages 9 and 15 y, a 2-point Likert scale (0 = 450 000, 1 = EPIC).
Saliva cell composition	At ages 9 and 15 y, the EpiDISH R package was developed for pediatric saliva epigenetic studies and estimated the proportion of epithelial cells, fibroblasts, and immune cells.^45^ These cells can influence changes and confound group differences in DNA methylation.^46,47^ Accounting for cell proportions improves the biological interpretability of estimates from analyses of EAA.^45^
City	Intraclass correlation coefficients in eTable 1 in Supplement 1 illustrated that a negligible amount of variance in our outcomes was attributable to city-level differences. Thus, parsimonious analyses were conducted with cities included as fixed effects as opposed to random effects.

Abbreviations: BMI, body mass index (calculated as weight in kilograms divided by height in meters squared); EAA, epigenetic age acceleration.

### Statistical Analysis

Using *Mplus* version 8.11 (Muthén and Muthén),^50^ we estimated 2 multilevel models with fixed and random effects. To test our first hypothesis with latent intercepts and slopes (ie, rate of change), our first multilevel model assigned time to level 1 and youth to level 2, which was justified by our intraclass correlation coefficients (see eTable 1 in Supplement 1 for the full sample and each ethnic and racial group). The intercept for each EAA measure was estimated by default at level 2. To examine changes in EAA over time in line with extant approaches that model a latent slope within a multilevel framework,^51^ we regressed each EAA measure on time at level 1, and this level 1 association was specified as a random slope at level 2, which was then regressed on all level 2 measures; this model included covariances between the intercepts and slopes to account for regression to the mean.^52^ For our second hypothesis and to understand the explanatory role of police intrusion, our second multilevel model regressed EAA measures at age 15 years on EAA measures at age 9 years, police intrusion, and our covariates, enabling us to examine the association between police intrusion and residualized changes in our EAA measures between ages 9 and 15 years. All models used bayesian estimation, which leverages posterior distributions to provide a more accurate representation of estimation uncertainty.^53,54^ Level 1 measures were person-mean centered, level 2 measures were grand-mean centered, and the Benjamini-Hochberg false discovery rate method was used to adjust for multiple hypotheses testing.^55^ Analyses were conducted from March 2023 to June 2024.

## Results

Among 2039 youths, the mean (SD) age at baseline was 9.27 (0.38) years; 1023 youths (50%) were male and 1016 (50%) were female; 917 (45%) were Black, 430 (21%) were Hispanic or Latino, 351 (17%) were White, and 341 (17%) were of another race or ethnicity, including 290 (85%) multiple and 51 (15%) self-identified other. Among youths in the Hispanic or Latino group, 89 (21%) self-identified as of Mexican descent, 65 (15%) as Central American, 10 (2%) as South American, and 266 (62%) with an overarching Hispanic or Latino identity.

### Descriptive Statistics

Zero-order bivariate correlations among key constructs are reported in eTable 3 in Supplement 1. Correlations among clocks illustrated that the Horvath clock had weakest correlations with the other clocks; the Hannum, GrimAge, and the DunedinPACE clocks had the strongest intercorrelations; and the PhenoAge clock was modestly correlated with the other clocks. These modest correlations among the diverse generations of epigenetic clocks have been attributable to the little overlap in CpG sites included in each clock’s algorithm.^19^ Correlations between each clock’s age 9- and 15-year assessments fell typically below 0.50, suggesting considerable interindividual variation in EAA change over time.

Paired-sample *t* tests for each EAA clock are reported in eTable 4 in Supplement 1, and changes in EAA for each ethnic and racial group are visualized in the eFigure in Supplement 1. White youths exhibited declines in EAA between ages 9 and 15 years in the Hannum, GrimAge, and DunedinPACE clocks, and Black, Hispanic or Latino, and other youths demonstrated no significant systematic linear rate of change in EAA over time.

### Stability and Change in EAA Between Childhood and Adolescence

Table 3 presents EAA intercepts and slopes regressed on youths’ ethnic and racial groups after adjusting for covariates (eTables 7-9 in Supplement 1). On average, Black youths showed lower initial EAA than White youths in the Horvath (B, −0.52; 95% CI, −0.83 to −0.21) and Hannum clocks (B, −0.80; 95% CI, −1.38 to −0.21), and Black, Hispanic/Latino, and other youths had higher initial EAA than White youths in the DunedinPACE clocks. Longitudinal results showed that White youths exhibited less accelerated EAA than did Black, Hispanic/Latino, and other youths from ages 9 to 15 years, particularly in the Hannum (B, 1.54; 95% CI, 0.36 to 2.18), GrimAge (B, 1.31; 95% CI, 0.68 to 1.97), and DunedinPACE epigenetic clocks (B, 0.27; 95% CI, 0.11 to 0.44).

**Table 3.** Baseline Bayesian Multilevel Model Examining Changes in Epigenetic Age Acceleration Among 2039 Youths Transitioning Between Childhood and Adolescence

Fixed effects	Intercept		Slope	
	Posterior estimate, mean (SD)	95% CI	Posterior estimate, mean (SD)	95% CI
**Horvath**				
Black (vs White)	−0.52 (0.16)^a^	−0.83 to −0.21	−0.24 (0.16)	−0.54 to 0.05
Hispanic/Latino (vs White)	−0.10 (0.19)	−0.49 to 0.27	−0.18 (0.18)	−0.54 to 0.17
Other (vs White)^b^	−0.14 (0.17)	−0.48 to 0.20	−0.15 (0.17)	−0.48 to 0.18
Intercepts	0.01 (0.05)	−0.08 to 0.10	0.05 (0.05)	−0.04 to 0.14
Random effects	3.83 (0.22)^a^	3.41 to 4.25	2.54 (0.70)^a^	1.43 to 3.68
**Hannum**				
Black (vs White)	−0.80 (0.30)^a^	−1.38 to −0.21	1.54 (0.49)^a^	0.36 to 2.18
Hispanic/Latino (vs White)	0.05 (0.36)	−0.66 to 0.76	0.90 (0.59)	−0.42 to 1.71
Other (vs White)^b^	−0.37 (0.32)	−1.00 to 0.25	0.71 (0.53)	−0.55 to 1.44
Intercepts	0.01 (0.09)	−0.16 to 0.18	−0.08 (0.14)	−0.36 to 0.19
Random effects	5.84 (0.81)^a^	4.39 to 7.57	2.52 (2.10)^a^	0.21 to 7.98
**GrimAge**				
Black (vs White)	0.29 (0.20)	−0.08 to 0.70	1.31 (0.33)^a^	0.68 to 1.97
Hispanic/Latino (vs White)	0.49 (0.25)	0.04 to 1.00	0.49 (0.38)	−0.21 to 1.24
Other (vs White)^b^	0.36 (0.21)	−0.06 to 0.80	0.67 (0.35)	−0.03 to 1.35
Intercepts	0.04 (0.06)	−0.08 to 0.15	−0.10 (0.08)	−0.27 to 0.06
Random effects	5.21 (0.46)^a^	4.68 to 6.49	8.86 (1.70)^a^	7.48 to 13.91
**PhenoAge**				
Black (vs White)	−0.41 (0.37)	−1.12 to 0.32	0.46 (0.41)	−0.34 to 1.29
Hispanic/Latino (vs White)	0.61 (0.45)	−0.27 to 1.48	0.19 (0.50)	−0.76 to 1.19
Other (vs White)^b^	−0.09 (0.39)	−0.85 to 0.69	0.10 (0.44)	−0.74 to 0.97
Intercepts	0.00 (0.11)	−0.21 to 0.21	0.09 (0.12)	−0.14 to 0.32
Random effects	17.67 (1.59)^a^	14.92 to 20.91	10.65 (5.46)	2.33 to 21.63
**DunedinPACE**				
Black (vs White)	0.27 (0.05)^a^	0.17 to 0.37	0.27 (0.09)^a^	0.11 to 0.44
Hispanic/Latino (vs White)	0.22 (0.06)^a^	0.10 to 0.35	0.12 (0.10)	−0.05 to 0.32
Other (vs White)^b^	0.13 (0.05)^a^	0.02 to 0.23	0.14 (0.09)	−0.04 to 0.31
Intercepts	0.00 (0.01)	−0.03 to 0.03	−0.01 (0.02)	−0.05 to 0.04
Random effects	0.27 (0.04)^a^	0.21 to 0.37	0.50 (0.16)^a^	0.31 to 0.86

^a^ Bayesian significance after accounting for multiple testing using the Benjamini-Hochberg false discovery rate method.

^b^ Other race and ethnicity groups included multiple races and self-identified other.

### The Role of Police Intrusion

Table 4 presents a multilevel model representing the association of police intrusion with youths’ ethnicity and race and their EAA after adjusting for covariates (eAppendix and eTables 10-11 in Supplement 1). On average, more Black youths reported police intrusion than White youths, and more police intrusion was associated with more EAA in the Hannum, GrimAge, PhenoAge, and DunedinPACE but not the Horvath clocks. The indirect effects via police intrusion were significant (Hannum: B, 0.11; 95% CI, 0.03-0.23; GrimAge: B, 0.09; 95% CI, 0.03-0.18; PhenoAge: B, 0.08; 95% CI, 0.02-0.18; DunedinPACE: B, 0.01; 95% CI, 0.01-0.03), suggesting that police intrusion was associated with differences in EAA between Black youths and White youths. However, police intrusion was not associated with differences in EAA among Hispanic or Latino youths, White youths, and youths of other races and ethnicities.

**Table 4.** Mediation Bayesian Multilevel Model Examining Residualized Changes in Epigenetic Age Acceleration Among 2039 Youths Transitioning Between Childhood and Adolescence via Police Intrusion

Fixed effects	Police intrusion, mean (SD; 95% CI)	Age-15 Horvath EAA measure, mean (SD; 95% CI)	Age-15 Hannum EAA measure, mean (SD; 95% CI)	Age-15 GrimAge EAA measure, mean (SD; 95% CI)	Age-15 PhenoAge EAA measure, mean (SD; 95% CI)	Age-15 DunedinPACE EAA measure, mean (SD; 95% CI)
Black (vs White)	0.25 (0.07; 0.12 to 0.40)^a^	−0.22 (0.16; −0.52 to 0.09)	0.94 (0.50; −0.01 to 1.90)	1.28 (0.31; 0.68 to 1.99)^a^	0.14 (0.38; −0.59 to 0.89)	0.37 (0.07; 0.23 to 0.52)^a^
Hispanic/Latino (vs White)	0.03 (0.09; −0.14 to 0.20)	−0.15 (0.16; −0.47 to 0.16)	0.48 (0.51; −0.49 to 1.46)	0.81 (0.32; 0.18 to 1.42)^a^	0.10 (0.40; −0.67 to 0.85)	0.20 (0.08; 0.05 to 0.35)^a^
Other (vs White)^b^	0.04 (0.07; −0.11 to 0.18)	−0.22 (0.19; −0.60 to 0.15)	0.81 (0.60; −0.35 to 1.97)	0.65 (0.38; −0.08 to 1.39)	0.39 (0.47; −0.51 to 1.30)	0.25 (0.09; 0.07 to 0.42)^a^
Police intrusion	NA	0.02 (0.05; −0.08 to 0.12)	0.44 (0.16; 0.13 to 0.76)^a^	0.36 (0.10; 0.17 to 0.56)^a^	0.35 (0.12; 0.11 to 0.59)^a^	0.06 (0.02; 0.01 to 0.11)^a^
Intercepts	0.00 (0.02; 0.00 to 0.02)	0.04 (0.05; −0.05 to 0.14)	0.04 (0.14; −0.31 to 0.25)	−0.03 (0.09; −0.20 to 0.16)	0.03 (0.11; −0.18 to 0.26)	−0.01 (0.02; −0.05 to 0.04)
Random effects	0.81 (0.03; 0.76 to 0.87)^a^	3.82 (0.12; 3.59 to 4.07)^a^	6.29 (1.19; 34.06 to 38.63)^a^	14.56 (0.47; 13.57 to 15.38)^a^	22.10 (0.72; 20.75 to 23.52)^a^	0.82 (0.03; 0.77 to 0.88)^a^

Abbreviation: NA, not applicable.

^a^ Bayesian significance after accounting for multiple testing using the Benjamini-Hochberg false discovery rate method.

^b^ Other race and ethnicity groups included multiple races and self-identified other.

### Sensitivity Analyses

To justify the temporal ordering between police intrusion and epigenetic aging, we tested whether the epigenetic clocks at age 9 years predicted police intrusion at age 15 years. As shown in eTable 12 in Supplement 1, neither EAA measure at age 9 years was associated with police intrusion at age 15 years. In addition, to rule out the possibility that the observed differences were attributable to technical issues with applying EAA algorithms to people of non-European ancestries, we examined correlations between epigenetic clocks and principal components of genomic ancestry within each ethnic and racial group. In eTables 13-17 in Supplement 1, the percentage of African genomic ancestry was uncorrelated with all clocks for Black youths, supporting the interpretation that the observed associations were linked to their lived experiences.

## Discussion

More US individuals of racially and ethnically minoritized groups are experiencing elevated disease risks than White US individuals, which may be attributable to accelerated biological aging following exposure to discrimination.^11^ The present cohort study quantified biological aging via EAA in a cohort spanning late childhood and midadolescence. Specifically, we tested whether EAA differed across ethnic and racial groups, whether these differences grew across development, and whether discriminatory encounters explained these differences. Longitudinal analyses showed EAA decelerated as youths matured; this change was more apparent for White youths than for their racially and ethnically minoritized peers across the 3 generations of epigenetic clocks, and racialized police intrusion was associated with these differences in EAA for Black youths.

Most Black and Hispanic or Latino youths and those of multiple or other races or ethnicities showed greater EAA in the second- and third-generation clocks. According to ecosocial,^15^ minority stress,^16^ and weathering hypotheses,^17^ social determinants of health related to racial discrimination may have explained associations between EAA and ethnicity and race. Indeed, as we found, police intrusive encounters occurred more frequently for Black youths than for White youths and, in turn, were associated with relative increases in EAA across the 3 generations of epigenetic clocks. In our sensitivity analysis, the clocks at age 9 years were not associated with police intrusion at age 15 years, providing support for the temporal ordering in our interpretations. Although police intrusion was not associated with differences among Hispanic or Latino, White, and other youths (who primarily reported multiple races), this result may be attributable to measurement, as the police intrusion scale was developed among a Black sample.^42^ Therefore, racialized experiences unique to Hispanic or Latino youths and those of multiple races or ethnicities were not captured and could not inform our statistical analyses, providing directions for future research to address.

Most Black, Hispanic or Latino, and multiple or other race or ethnicity youths did not show decreases in epigenetic aging over time as did their White peers. Consistent with life course theory,^30^ discrimination can inhibit healthy development during sensitive periods, including the transition from childhood to adolescence. Structural factors, such as socioeconomic and neighborhood disadvantage, can limit ethnically and racially minoritized youths’ access to health-promoting resources.^5^ In addition, the correlations between each EAA measure across waves did not suggest that EAA was stable across time, supporting prior research that found nonlinear growth during adolescence.^56^

There was a notable pattern that showed Black youths had lower initial EAA than their White peers in the first-generation (ie, Horvath and Hannum) clocks. However, Black youths showed increases in EAA over time relative to White youths in the Hannum clock. This finding is reminiscent of a pattern found in telomere length, wherein Black individuals had longer (ie, more favorable) telomeres at birth but faster (ie, more unfavorable) telomere length shortening over time than White individuals.^57^ A similar pattern was found in a cross-sectional sample of Black and White adults, wherein telomeres were longer for Black adults aged 19 to 79 years but shorter for Black adults aged 80 years and older.^58^ These age-related differences suggest that our results for the Horvath and Hannum clocks in the present study were not regression artifacts. Thus, the present study adds to the literature finding that Black individuals exhibit lower EAA initially^18,19^ but subsequently experience faster EAA over time—a pattern possibly obscured by cross-sectional analyses.

Notably, White youths showed decreases in EAA over time. In line with life course theory, healthy development is not strictly linear but is in a constant state of flux due to diverse influences at different points in life.^30^ This flux may be attributable to events between 2007 and 2017, characterized by an economic recession followed by an economic recovery. This economic recovery benefited more White families than ethnically and racially minoritized famlies,^59^ providing some contextual indication that this improvement may have explained why decreased EAA emerged for White youths. Consistent with prior research,^60^ White youths showed telomere lengthening (eg, reverse biological aging) during this period.

### Limitations

The present study has several limitations. First, merging small ethnic and racial subgroups into an overarching other category may have removed significant group heterogeneity; moreover, each group had small sample sizes, making it difficult to detect subgroup differences. Second, although we included covariances between the latent intercepts and slopes, which is a recommended practice to adjust for possible regression to the mean,^52^ we did not have baseline EAA data in addition to that at ages 9 and 15 years to fully rule this out. Third, the present study was a correlational analysis; therefore, neither our results nor conclusions are causally definitive.

## Conclusions

As scholars conceptualize associations among EAA, mortality, and morbidity,^9,61^ future research should consider effective strategies that mitigate EAA among ethnically and racially minoritized communities. While some scholars suggest that resilience may be a key mechanism to helping ethnically and racially minoritized communities cope with ethnic and racial discrimination, more research is needed about how to effectively dismantle policies and institutions, such as biased policing in law enforcement, that contribute to and perpetuate discrimination against historically marginalized individuals.^62^ By elucidating possible health implications of racial or ethnic disparities in EAA, we aim to spur engagement with and support for disparity-mitigating policies and practices.^63^ The health and well-being of the US relies on health equity across all ethnic and racial groups.

## Notes

Supplement 1. eAppendix eFigure. Trajectories of Epigenetic Age Acceleration Between Ages 9 and 15 with 95% Confidence Intervals for Each Epigenetic Age Clock eTable 1. Intraclass Correlation Coefficients eTable 2. Zero-Order Bivariate Correlations Between Maternal/Paternal Education at Children’s Birth and Within-Person Changes in Epigenetic Age Acceleration Between Children’s Ages 9 and 15 eTable 3. Zero-Order Bivariate Correlations among All Study Measures eTable 4. Paired Samples-Tests of Epigenetic Age Acceleration eTable 5. Posterior Estimates (SD) from a Bayesian Multilevel Model Examining DNA Methylation-Age among 2,039 Youth Transitioning Between Childhood and Adolescence eTable 6. Posterior Estimates (SD) from a Bayesian Multilevel Model Examining Changes in DNA Methylation-Age among 2,039 Youth Transitioning Between Childhood and Adolescence eTable 7. Posterior Estimates (SD) from a Bayesian Multilevel Model Examining Changes in Epigenetic Age Acceleration among 2,039 Youth Transitioning Between Childhood and Adolescence eTable 8. Posterior Estimates (SD) from a Bayesian Multilevel Model Examining Changes in Epigenetic Age Acceleration among 2,039 Youth Transitioning Between Childhood and Adolescence eTable 9. Posterior Estimates (SD) from a Bayesian Multilevel Model Examining Changes in Epigenetic Age Acceleration among 2,039 Youth Transitioning Between Childhood and Adolescence eTable 10. Posterior Estimates (SD) from a Bayesian Multilevel Model Examining Residualized Changes in Epigenetic Age Acceleration among 2,039 Youth Transitioning Between Childhood and Adolescence eTable 11. Posterior Estimates (SD) from a Bayesian Multilevel Model Examining Residualized Changes in Epigenetic Age Acceleration among 2,039 Youth Transitioning Between Childhood and Adolescence eTable 12. Posterior Estimates (SD) from a Bayesian Multilevel Model Examining Main Effects on Age-15 Direct Police Intrusion among 2,039 Youth eTable 13. Zero-Order Bivariate Correlations Between Principal Components (PCs) and the Horvath Epigenetic Age Acceleration Measure at Ages 9 and 15 among 2,039 Youth eTable 14. Zero-Order Bivariate Correlations Between Principal Components (PCs) and the Hannum Epigenetic Age Acceleration Measure at Ages 9 and 15 among 2,039 Youth eTable 15. Zero-Order Bivariate Correlations Between Principal Components (PCs) and the GrimAge Epigenetic Age Acceleration Measure at Ages 9 and 15 among 2,039 Youth eTable 16. Zero-Order Bivariate Correlations Between Principal Components (PCs) and the PhenoAge Epigenetic Age Acceleration Measure at Ages 9 and 15 among 2,039 Youth eTable 17. Zero-Order Bivariate Correlations Between Principal Components (PCs) and the DunedinPACE Epigenetic Age Acceleration Measure at Ages 9 and 15 among 2,039 Youth

Supplement 2. Data sharing statement
