# Supplementary material for: Longitudinal Changes in Epigenetic Age Acceleration Across Childhood and Adolescence
Source: JAMA Pediatr. 2024 Oct 7;178(12):1298–306. doi: 10.1001/jamapediatrics.2024.3669 (PMC11459359; doi:10.1001/jamapediatrics.2024.3669)
Supplement: Supplement 1. — eAppendix eFigure. Trajectories of Epigenetic Age Acceleration Between Ages 9 and 15 with 95% Confidence Intervals for Each Epigenetic Age Clock eTable 1. Intraclass Correlation Coefficients eTable 2. Zero-Order Bivariate Correlations Between Maternal/Paternal Education at Children’s Birth and Within-Person Changes in Epigenetic Age Acceleration Between Children’s Ages 9 and 15 eTable 3. Zero-Order Bivariate Correlations among All Study Measures eTable 4. Paired Samples-Tests of Epigenetic Age Acceleration eTable 5. Posterior Estimates (SD) from a Bayesian Multilevel Model Examining DNA Methylation-Age among 2,039 Youth Transitioning Between Childhood and Adolescence eTable 6. Posterior Estimates (SD) from a Bayesian Multilevel Model Examining Changes in DNA Methylation-Age among 2,039 Youth Transitioning Between Childhood and Adolescence eTable 7. Posterior Estimates (SD) from a Bayesian Multilevel Model Examining Changes in Epigenetic Age Acceleration among 2,039 Youth Transitioning Between Childhood and Adolescence eTable 8. Posterior Estimates (SD) from a Bayesian Multilevel Model Examining Changes in Epigenetic Age Acceleration among 2,039 Youth Transitioning Between Childhood and Adolescence eTable 9. Posterior Estimates (SD) from a Bayesian Multilevel Model Examining Changes in Epigenetic Age Acceleration among 2,039 Youth Transitioning Between Childhood and Adolescence eTable 10. Posterior Estimates (SD) from a Bayesian Multilevel Model Examining Residualized Changes in Epigenetic Age Acceleration among 2,039 Youth Transitioning Between Childhood and Adolescence eTable 11. Posterior Estimates (SD) from a Bayesian Multilevel Model Examining Residualized Changes in Epigenetic Age Acceleration among 2,039 Youth Transitioning Between Childhood and Adolescence eTable 12. Posterior Estimates (SD) from a Bayesian Multilevel Model Examining Main Effects on Age-15 Direct Police Intrusion among 2,039 Youth eTable 13. Zero-Order Bivariate Correlations Between Principal [file jamapediatr-e243669-s001.pdf]

Supplemental Online Content

Del Toro J, Martz C, Freilich CD, et al. Longitudinal changes in epigenetic age acceleration across childhood and adolescence. *JAMA Pediatr*. Published online October 7, 2024. doi:10.1001/jamapediatrics.2024.3669

eAppendix
eFigure. Trajectories of Epigenetic Age Acceleration Between Ages 9 and 15 with 95% Confidence Intervals for Each Epigenetic Age Clock
eTable 1. Intraclass Correlation Coefficients
eTable 2. Zero-Order Bivariate Correlations Between Maternal/Paternal Education at Children's Birth and Within-Person Changes in Epigenetic Age Acceleration Between Children's Ages 9 and 15
eTable 3. Zero-Order Bivariate Correlations among All Study Measures
eTable 4. Paired Samples-Tests of Epigenetic Age Acceleration
eTable 5. Posterior Estimates (SD) from a Bayesian Multilevel Model Examining DNA Methylation-Age among 2,039 Youth Transitioning Between Childhood and Adolescence
eTable 6. Posterior Estimates (SD) from a Bayesian Multilevel Model Examining Changes in DNA Methylation-Age among 2,039 Youth Transitioning Between Childhood and Adolescence
eTable 7. Posterior Estimates (SD) from a Bayesian Multilevel Model Examining Changes in Epigenetic Age Acceleration among 2,039 Youth Transitioning Between Childhood and Adolescence
eTable 8. Posterior Estimates (SD) from a Bayesian Multilevel Model Examining Changes in Epigenetic Age Acceleration among 2,039 Youth Transitioning Between Childhood and Adolescence
eTable 9. Posterior Estimates (SD) from a Bayesian Multilevel Model Examining Changes in Epigenetic Age Acceleration among 2,039 Youth Transitioning Between Childhood and Adolescence
eTable 10. Posterior Estimates (SD) from a Bayesian Multilevel Model Examining Residualized Changes in Epigenetic Age Acceleration among 2,039 Youth Transitioning Between Childhood and Adolescence
eTable 11. Posterior Estimates (SD) from a Bayesian Multilevel Model Examining Residualized Changes in Epigenetic Age Acceleration among 2,039 Youth Transitioning Between Childhood and Adolescence
eTable 12. Posterior Estimates (SD) from a Bayesian Multilevel Model Examining Main Effects on Age-15 Direct Police Intrusion among 2,039 Youth
eTable 13. Zero-Order Bivariate Correlations Between Principal Components (PCs) and the Horvath Epigenetic Age Acceleration Measure at Ages 9 and 15 among 2,039 Youth

© 2024 Del Toro J et al. *JAMA Pediatrics.*

eTable 14. Zero-Order Bivariate Correlations Between Principal Components (PCs) and the Hannum Epigenetic Age Acceleration Measure at Ages 9 and 15 among 2,039 Youth
eTable 15. Zero-Order Bivariate Correlations Between Principal Components (PCs) and the GrimAge Epigenetic Age Acceleration Measure at Ages 9 and 15 among 2,039 Youth
eTable 16. Zero-Order Bivariate Correlations Between Principal Components (PCs) and the PhenoAge Epigenetic Age Acceleration Measure at Ages 9 and 15 among 2,039 Youth
eTable 17. Zero-Order Bivariate Correlations Between Principal Components (PCs) and the DunedinPACE Epigenetic Age Acceleration Measure at Ages 9 and 15 among 2,039 Youth

This supplemental material has been provided by the authors to give readers additional information about their work.

© 2024 Del Toro J et al. *JAMA Pediatrics.*

eAppendix

Procedure and Exclusionary Criteria

The FFCW study randomly sampled 4,898 births from 75 hospitals in 20 large cities between 1998 and 2000 with an oversample of nonmarital births (at a ratio of 3:1). Mothers and fathers were interviewed soon after the birth of the focal child, and follow-up interviews were conducted when children were approximately ages 1 (1999–2001), 3 (2001–2003), 5 (2003–2006), 9 (2007–2010), 15 (2014-2017), and 22 (2020-2024). The present study primarily used waves of data when children were on average ages 9 and 15, which were the only waves when saliva samples were collected and currently available to assess children's epigenetic profiles. Nonetheless, we used baseline and age-22 data for descriptive purposes and to strengthen the present study's inferences and methodology. The University of [blinded for review] Institutional Review Board (IRB) deemed the present study was not human subjects research as we analyzed de-identified, publicly available data [exempt Criteria 45-CFR-46.104(d)(4)].

Between the excluded (n=2,859) and included samples of children (n=2,039), the included sample was more likely to be White [than Latinx; $\chi^2(3)=10.91$, $p<.05$], female [than male; $\chi^2(1)=5.73$, $p<.05$], have a mother with a more advanced degree [excluded: M=2.45, SD=0.99; included: M=2.52, SD=1.00; $t(2788)=7.24$, $p<.001$], to be chronologically younger [excluded: M=9.36, SD=0.41; included: M=9.26, SD=0.38; $t(3511)=2.15$, $p<.05$], but did not differ by BMI z-scores [$t(3127)=1.36$, $p=.17$], smoking [$\chi^2(1)=1.77$, $p=.18$].

Participants: Demographic Information

In Table 1 of the main manuscript, Latinx children were, on average, chronologically older in age than their Black, White, and Other peers, who did not differ from each other. On average, White children lived in households where mothers had the most advanced educational

degrees, Latinx children lived in households where mothers had the least advanced degrees, and Black and Other children did not differ from each other. On average, White youth had smaller body mass index (BMI) z-scores than youth of color, who did not significantly differ from each other in their BMI z-scores. No significant ethnic/racial group differences emerged for children's sex and smoking.

Validity of EAA among Youth from Diverse Ethnic/Racial Groups

We assessed criterion-related (i.e., concurrent, parallel, and predictive) validity of the epigenetic clocks among our ethnically/racially diverse sample of youth. Because the first-generation DNA methylation-age measures (i.e., Horvath and Hannum) were developed to predict chronological age and the second- and third-generation versions (i.e., GrimAge, PhenoAge, and DunedinPACE) were trained to predict health-related outcomes among adults, we tested whether the first- and second-/third- generation clocks were linked to youth's chronological age and self-rated health, respectively. In doing so, we estimated three unconditional two-level multilevel models; in the first model, we regressed the time-varying first-generation DNA methylation-age clocks on chronological age; in the second model, we regressed time-varying self-rated health on time-varying second- and third- generation EAA measures; these two models were leveraged to provide evidence of concurrent validity. Thereafter, to provide evidence of predictive validity, the third multilevel model regressed youth's chronological age and self-rated health at age 22 on our time-varying first-generation DNA methylation age clocks and our time-varying second- and third-generation EAA measures, respectively. Following each of the aforementioned multilevel models, to provide evidence of parallel validity, we used multi-group analyses with children's ethnicity/race as the grouping

variable to assess whether such links could be constrained to be equivalent across ethnic/racial groups without causing a significant decrement in model fit.

Turning first to the first-generation epigenetic clocks, we tested whether the first-generation DNA methylation-age clocks (i.e., Horvath and Hannum, which were not detrended for chronological age) were related to chronological age for the full sample and comparably among the four ethnic/racial groups. For the full sample, we found that the raw Horvath and Hannum time-varying clocks were related to time-varying chronological age (Horvath: β=0.81, *SE*=0.13, p<.001; Hannum: β=0.40, *SE*=0.09, p<.001). After subsequent multi-group tests, we found that we could constrain the time-varying links between children's first-generation clocks and their chronological age to be equivalent across the four ethnic/racial groups without causing a significant decrement in model fit, $\Delta\chi^2(6)$=12.31, p=.06. Therefore, these analyses yielded that the first-generation clocks showed evidence of concurrent and parallel validity among our ethnically/racially diverse sample of youth.

For the second-generation clock (i.e., PhenoAge and GrimAge clocks, which were detrended for age) and the third-generation clock (i.e., DunedinPACE), we tested whether these EAA measures concurrently were associated with self-rated health for the full sample and comparably among the four ethnic/racial groups. For the full sample, time-varying self-rated health was correlated with less time-varying EAA measures (GrimAge: β=-0.04, *SE*=0.02, p<.05; PhenoAge: β =-0.05, *SE*=0.02, p=.01; DunedinPACE: β =-0.08, *SE*=0.02, p<.001). Next, our multi-group analysis illustrated that we could constrain these parameters to be equivalent across the four ethnic/racial groups without causing a significant decrement in model fit, $\Delta\chi^2(9)$=5.97, p=.74. Thus, these analyses yielded that the second- and third-generation clocks

© 2024 Del Toro J et al. *JAMA Pediatrics.*

showed evidence of concurrent and parallel validity among our ethnically/racially diverse sample of youth.

Turning to our predictive-validity assessments, we found that the first-generation DNA methylation-age measures were linked to youth's age-22 chronological age (Horvath: β=0.02, *SE*=0.01, p<.01; Hannum: β=0.01, *SE*=0.00, p<.05), and constraining these parameters to be equivalent across ethnic/racial groups did not result in a significant decrement in model fit, $\Delta\chi^2(6)$=3.19, p=.78. In addition, the second- and third-generation clocks were associated with youth's age-22 self-rated health (GrimAge: β=-0.02, *SE*=0.01, p<.05; PhenoAge: β =-0.02, *SE*=0.01, p<.01; DunedinPACE: β =-0.30, *SE*=0.11, p<.01), and constraining these parameters to be equivalent across ethnic/racial groups did not result in a significant decrement in model fit, $\Delta\chi^2(9)$=14.99, p=.09. Altogether, in line with our prior analyses and in addition to showing evidence of parallel validity, we also found evidence of predictive validity for our epigenetic clocks.

Overall, these results provide evidence that the DNA methylation-age and EAA measures demonstrated measurement criterion-related validity across the multiple ethnic/racial groups.

Missing Data

Among the 2,039 children and 20 key constructs, 1,691 (or 83% of) children had complete data on all measures; 211 (10%) had one variable missing; four (0.2%) had two variables missing; 115 (6%) had six variables missing; 10 (0.5%) had seven variables missing; three (0.1%) had nine variables missing; and five (0.2%) had 11 variables missing. The count of missing variables was unrelated to children's ethnicity/race (r-range for categorical ethnicity/race variables=-.01 to .04, p-range=.08 to .84), sex (r=.01, p=.65), age (r=.01, p=.68), maternal education (r=-.02, p=.41), and BMI z-scores (r=.02, p=.32). After adjusting for covariates, semi-

partial correlations showed that the count of missingness was unrelated to the Horvath (age-9: r=.02, p=.49; age-15: r=.02, p=.32), Hannum (age-9: r=.00, p=.94; age-15: r=-.01, p=.72), GrimAge (age-9: r=.00, p=.99; age-15: r=-.01, p=.76), PhenoAge (age-9: r=.00, p=.92; age-15: r=.02, p=.50), and DunedinPACE epigenetic clocks (age-9: r=.00, p=.96; age-15: r=.00, p=.92), characterizing our data as conditionally missing at random.[1]

Sensitivity Analysis: **Genetic Ancestry**

With DNA sequence variation varying by genetic ancestry, we examined correlations between epigenetic clocks and principal components (PCs) of genomic ancestry within each ethnic/racial group. In eTables 13-17, within-analytic group PCs 1-20 are included for each ethnic/racial group (White youth: pc1e-pc20e; Black youth: pc1a-pc20a; Latinx and Other youth of color: pc1h-pc20h). The last letter for the aforementioned variables (i.e., a, e, or h) indicates which ancestry the group is included in that variable: a – predominantly African analytic group, e – predominantly European analytic group, h – predominantly Hispanic analytic group. The PCs control for any genetic aspects of common ancestry that may spuriously correlate with outcomes of interest.[2]

© 2024 Del Toro J et al. *JAMA Pediatrics.*

References

1. Enders CK. Missing data: An update on the state of the art. *Psychological Methods*. Published online 2023. doi:10.1037/met0000563

2. Price AL, Patterson NJ, Plenge RM, Weinblatt ME, Shadick NA, Reich D. Principal components analysis corrects for stratification in genome-wide association studies. *Nature Genetics*. 2006;38(8):904-909. doi:10.1038/ng1847

© 2024 Del Toro J et al. *JAMA Pediatrics.*

eFigure

Trajectories of Epigenetic Age Acceleration Between Ages 9 and 15 with 95% Confidence Intervals for Each Epigenetic Age Clock

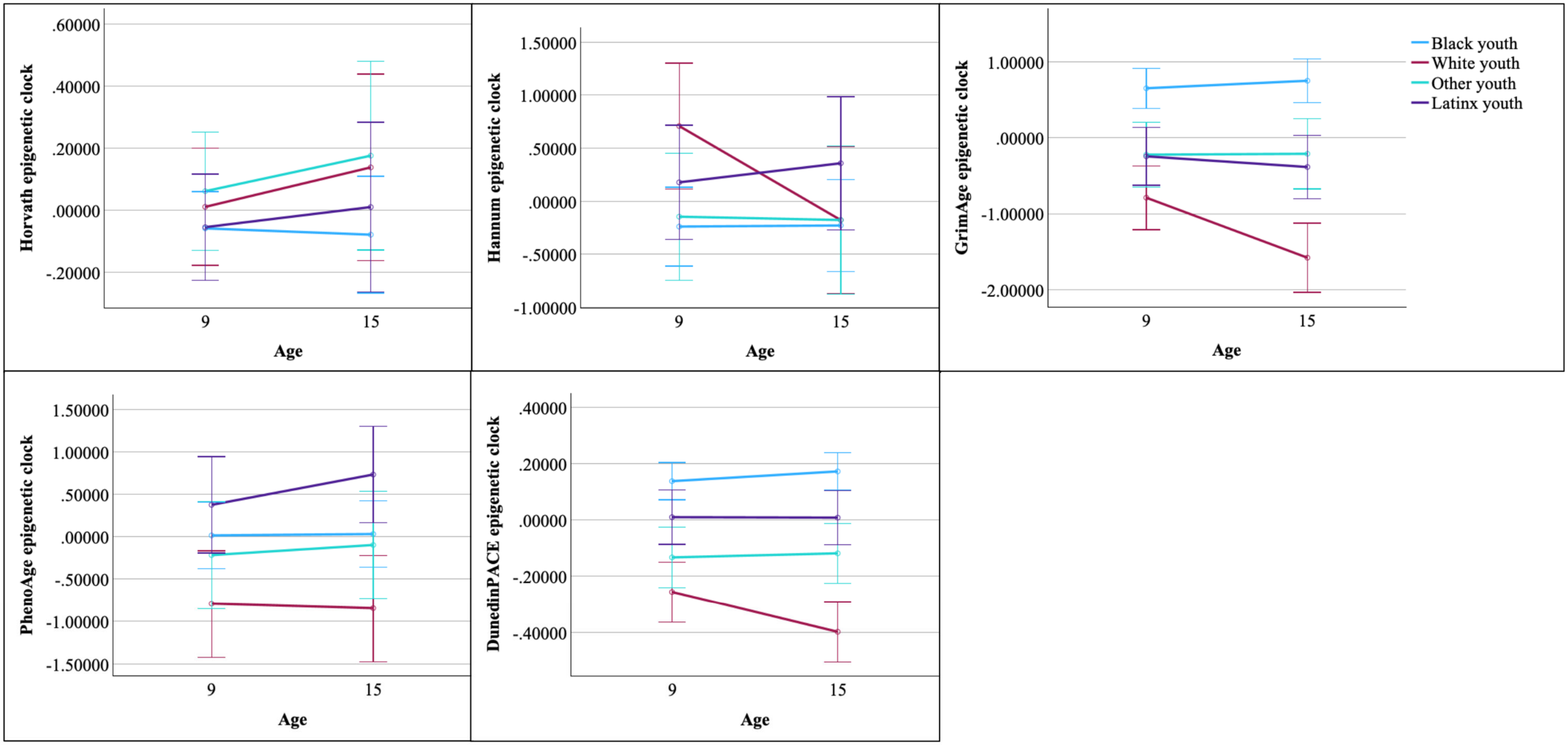

© 2024 Del Toro J et al. *JAMA Pediatrics.*

eTable 1

Intraclass Correlation Coefficients

Epigenetic clocks	Proportion of variance explained by within-person differences	Proportion of variance explained by between-person differences	Proportion of variance explained by between-city differences
Full sample			
Horvath	0.35	0.65	0.00
Hannum	0.69	0.31	0.00
GrimAge	0.58	0.41	0.01
PhenoAge	0.43	0.56	0.01
DunedinPACE	0.67	0.32	0.01
Black youth			
Horvath	0.31	0.67	0.02
Hannum	0.69	0.31	0.00
GrimAge	0.55	0.45	0.00
PhenoAge	0.39	0.61	0.00
DunedinPACE	0.68	0.32	0.00
White youth			
Horvath	0.31	0.67	0.02
Hannum	0.47	0.53	0.00
GrimAge	0.68	0.29	0.03
PhenoAge	0.55	0.43	0.02
DunedinPACE	0.71	0.28	0.01
Latinx youth			
Horvath	0.36	0.64	0.00
Hannum	0.76	0.24	0.00
GrimAge	0.65	0.35	0.00
PhenoAge	0.46	0.53	0.01
DunedinPACE	0.70	0.29	0.01
Other youth			
Horvath	0.34	0.65	0.01

© 2024 Del Toro J et al. *JAMA Pediatrics.*

Hannum	0.69	0.31	0.00
GrimAge	0.61	0.38	0.01
PhenoAge	0.44	0.53	0.03
DunedinPACE	0.73	0.26	0.01

© 2024 Del Toro J et al. *JAMA Pediatrics.*

eTable 2

Zero-Order Bivariate Correlations Between Maternal/Paternal Education at Children's Birth and Within-Person Changes in Epigenetic Age Acceleration Between Children's Ages 9 and 15

Parental education at birth	Horvath	Hannum	PhenoAge	GrimAge	DunedinPACE
Maternal education					
Moms with less than high school degree (vs. everyone else)	-.01	.00	.04**	.03*	.06**
Moms with a high school degree (vs. everyone else)	.01	-.02	.01	.03	.00
Moms with some college or technical degree (vs. everyone else)	.02	.00	-.02	-.01	-.02
Moms with a college or more advanced degree (vs. everyone else)	-.02	.03	-.06**	-.07**	-.08**
Paternal education					
Dads with less than high school degree (vs. everyone else)	.01	-.02	.04*	.03*	.05**
Dads with a high school degree (vs. everyone else)	.01	-.01	.02	.03	.03*
Dads with some college or technical degree (vs. everyone else)	-.02	.01	-.02	-.03	-.04**
Dads with a college or more advanced degree (vs. everyone else)	-.01	.03*	-.06*	-.06**	-.07**

Note: * $p < .05$, ** $p < .01$.

© 2024 Del Toro J et al. *JAMA Pediatrics.*

eTable 3

Zero-Order Bivariate Correlations among All Study Measures

	Variables	1	2	3	4	5	6	7	8	9	10	11	12	13	14	15	16	171	18	19	20	21	22	23	24
1	Black child	1																							
2	Other child	**-.14**	1																						
3	Latinx child	**-.47**	**-.08**	1																					
4	White child	**-.41**	**-.07**	**-.24**	1																				
5	Police intrusion	**.14**	-.02	-.04	**-.10**	1																			
6	Female child	.01	-.04	.01	-.01	**-.21**	1																		
7	Child age	**-.05**	.00	**.13**	**-.08**	.01	.03	1																	
8	Family SES	**-.20**	**.10**	**-.19**	**.38**	**-.15**	-.03	**-.08**	1																
9	Child's smoking	-.01	-.02	-.01	.02	.04	-.04	-.02	-.04	1															
10	Maternal smoking	.02	-.02	**-.15**	**.12**	**.09**	.01	.01	**-.16**	.03	1														
11	DNAm smoking	**.36**	**-.05**	**-.17**	**-.24**	.04	.01	-.02	**-.15**	.01	**.07**	1													
12	Child BMI	**.06**	.00	**.07**	**-.14**	-.01	**.05**	.04	**-.11**	.03	-.01	.04	1												
13	Child puberty	**.08**	.00	-.02	**-.09**	.01	**.11**	.01	-.04	.02	-.01	.00	**-.26**	1											
14	Epic (vs. 450K)	**-.08**	.03	**.07**	.00	.01	.01	.02	**-.05**	.02	**.05**	.00	-.03	.02	1										
15	Age-9 Horvath EAA	-.02	-.02	-.01	.01	**-.05**	.02	.02	.03	-.01	-.03	**.21**	**.07**	**-.05**	.00	1									
16	Age-15 Horvath EAA	-.03	-.01	.00	.02	-.03	-.01	-.03	.01	.02	-.01	**.17**	**.09**	-.04	.00	**.72**	1								
17	Age-9 Hannum EAA	**-.05**	-.02	.01	**.07**	-.01	**-.05**	.01	**.06**	-.03	**-.05**	-.02	**.05**	-.03	.00	**.30**	**.19**	1							
18	Age-15 Hannum EAA	-.01	.01	.03	-.01	**.07**	**-.07**	.02	.01	.02	-.01	**-.06**	.01	-.01	.00	.13	.20	.31	1						
19	Age-9 GrimAge EAA	**.13**	**-.06**	-.04	**-.10**	**.06**	**-.15**	.00	**-.05**	-.02	-.04	**.37**	**.09**	-.01	.00	**.32**	**.22**	**.68**	**.14**	1					
20	Age-15 GrimAge EAA	**.17**	-.02	-.04	**-.16**	**.12**	**-.07**	-.02	**-.10**	.01	.00	**.24**	**.10**	-.02	.00	**.21**	**.26**	**.20**	**.71**	**.42**	1				
21	Age-9 Phenoage EAA	.01	-.03	.04	**-.05**	-.04	**.14**	.02	**-.07**	-.02	.00	**.17**	**.13**	-.02	.00	**.44**	**.32**	**.49**	**.12**	**.44**	**.18**	1			
22	Age-15 Phenoage EAA	.01	.01	**.06**	**-.06**	.00	**.19**	.02	**-.07**	-.02	.01	**.07**	**.13**	-.03	.00	**.29**	**.40**	**.21**	**.59**	**.18**	**.48**	**.56**	1		
23	Age-9 DunedinPACE EAA	**.12**	**-.06**	.01	**-.11**	.03	.01	.00	**-.09**	-.01	**-.06**	.04	**.19**	**-.07**	.00	**.08**	.01	**.67**	**.14**	**.67**	**.22**	**.41**	**.15**	1	
24	Age-15 DunedinPACE EAA	**.16**	-.02	.01	**-.17**	**.06**	**.13**	-.01	**-.12**	.04	.00	.01	**.16**	**-.07**	.00	.02	.03	**.14**	**.69**	**.15**	**.70**	**.11**	**.43**	**.32**	1

Note: Bolded values suggest correlations with *p*-values < .05; non-bolded values indicate correlations with *p*-values ≥ .05. *EAA* = Epigenetic Age Acceleration, *DNAm* = DNA methylation

© 2024 Del Toro J et al. *JAMA Pediatrics.*

eTable 4

Paired Samples-Tests of Epigenetic Age Acceleration

Epigenetic clocks	Age 9: *M (SD)*	Age 15: *M (SD)*	Paired samples t-tests
Full sample (n = 2,039)			
Horvath	-0.01 (1.75)	0.04 (2.79)	$t(1905) = -1.06, p = .29$
Hannum	-0.01 (5.51)	-0.14 (6.41)	$t(1905) = 0.82, p = .41$
GrimAge	-0.01 (3.93)	-0.13 (4.32)	$t(1905) = 1.18, p = .24$
PhenoAge	-0.04 (5.82)	0.04 (5.81)	$t(1905) = -0.69, p = .49$
DunedinPACE	-0.01 (1.00)	-0.01 (1.00)	$t(1905) = 0.22, p = .82$
Black (n = 917)			
Horvath	-0.05 (1.83)	-0.06 (2.87)	$t(846) = 0.24, p = .81$
Hannum	-0.28 (5.99)	-0.29 (6.99)	$t(846) = 0.04, p = .96$
GrimAge	0.58 (4.26)	0.66 (4.51)	$t(846) = -0.55, p = .58$
PhenoAge	0.06 (6.46)	0.08 (6.26)	$t(846) = -0.07, p = .94$
DunedinPACE	0.13 (1.03)	0.16 (1.00)	$t(846) = -0.70, p = .49$
White (n = 351)			
Horvath	0.02 (1.48)	0.15 (2.62)	$t(331) = -1.10, p = .27$
Hannum	0.67 (5.33)	-0.21 (5.77)	$t(331) = 2.57, p < .05$
GrimAge	-0.86 (3.62)	-1.63 (4.03)	$t(331) = 3.16, p < .01$
PhenoAge	-0.74 (5.22)	-0.82 (5.69)	$t(331) = 0.23, p = .81$
DunedinPACE	-0.26 (0.98)	-0.38 (0.98)	$t(331) = 1.95, p = .05$
Other youth of color (n = 341)			
Horvath	0.07 (1.79)	0.18 (2.72)	$t(324) = -1.06, p = .29$
Hannum	-0.18 (4.90)	-0.22 (6.07)	$t(324) = 0.11, p = .91$
GrimAge	-0.29 (3.64)	-0.27 (4.10)	$t(324) = -0.09, p = .93$
PhenoAge	-0.17 (5.49)	-0.06 (5.51)	$t(324) = -0.38, p = .71$
DunedinPACE	-0.13 (0.94)	-0.11 (0.96)	$t(324) = -0.34, p = .74$
Latinx (n = 430)			
Horvath	-0.05 (1.75)	0.02 (2.84)	$t(401) = -0.65, p = .51$
Hannum	0.14 (5.02)	0.30 (5.89)	$t(401) = -0.48, p = .63$
GrimAge	-0.31 (3.51)	-0.45 (3.69)	$t(401) = 0.69, p = .49$

PhenoAge	0.42 (5.05)	0.77 (5.04)	*t*(401) = -1.45, *p* = .15
DunedinPACE	0.01 (0.93)	0.00 (0.94)	*t*(401) = 0.02, *p* = .99

© 2024 Del Toro J et al. *JAMA Pediatrics.*

eTable 5

Posterior Estimates (SD) from a Bayesian Multilevel Model Examining DNA Methylation-Age among 2,039 Youth Transitioning Between Childhood and Adolescence

	Horvath				Hannum			
	Intercept		Slope		Intercept		Slope	
Measures	*Estimate (SD)*	*95% CI*	*Estimate (SD)*	*95% CI*	*Estimate (SD)*	*95% CI*	*Estimate (SD)*	*95% CI*
Fixed effects								
Black (vs. White)	-0.53 (0.16)*	[-0.85, -0.21]	-0.25 (0.16)	[-0.56, 0.06]	-0.79 (0.30)*	[-1.37, -0.20]	1.55 (0.49)*	[0.58, 2.50]
Other (vs. White)	-0.14 (0.17)	[-0.49, 0.19]	-0.13 (0.17)	[-0.48, 0.21]	-0.36 (0.32)	[-0.99, 0.26]	0.72 (0.53)	[-0.33, 1.74]
Latinx (vs. White)	-0.11 (0.20)	[-0.50, 0.27]	-0.21 (0.20)	[-0.58, 0.18]	0.06 (0.36)	[-0.65, 0.77]	0.91 (0.59)	[-0.24, 2.07]
Female (vs. male)	0.06 (0.09)	[-0.13, 0.24]	-0.14 (0.09)	[-0.33, 0.05]	-0.62 (0.18)*	[-0.96, -0.27]	-0.58 (0.28)	[-1.14, -0.02]
Child age	0.36 (0.14)	[0.09, 0.64]	-0.55 (0.14)*	[-0.83, -0.26]	0.76 (0.26)*	[0.26, 1.28]	-0.80 (0.42)	[-1.63, 0.04]
Family SES	0.04 (0.06)	[-0.09, 0.16]	-0.07 (0.06)	[-0.19, 0.05]	-0.05 (0.12)	[-0.27, 0.18]	0.02 (0.19)	[-0.35, 0.39]
Child smoking	0.16 (0.49)	[-0.80, 1.12]	0.57 (0.52)	[-0.45, 1.57]	0.43 (0.91)	[-1.34, 2.23]	2.26 (1.50)	[-0.68, 5.29]
Maternal smoking	-0.18 (0.12)	[-0.42, 0.07]	0.10 (0.12)	[-0.15, 0.34]	0.00 (0.23)	[-0.46, 0.45]	0.30 (0.38)	[-0.44, 1.04]
DNAm smoking	0.51 (0.05)*	[0.41, 0.61]	0.13 (0.05)*	[0.03, 0.23]	-0.11 (0.10)	[-0.29, 0.08]	-0.55 (0.15)*	[-0.85, -0.24]
Child BMI	0.16 (0.04)*	[0.07, 0.24]	0.12 (0.04)*	[0.03, 0.20]	0.22 (0.08)*	[0.06, 0.38]	-0.04 (0.13)	[-0.30, 0.21]
Child puberty	-0.10 (0.09)	[-0.28, 0.07]	-0.04 (0.09)	[-0.22, 0.13]	0.06 (0.16)	[-0.27, 0.37]	-0.04 (0.27)	[-0.56, 0.50]
EPIC (vs. 450K)	0.02 (0.10)	[-0.18, 0.23]	-0.12 (0.10)	[-0.32, 0.09]	0.22 (0.20)	[-0.17, 0.60]	-0.49 (0.32)	[-1.11, 0.12]
Prop. of epithelial cells	0.38 (7.31)	[-13.93, 14.65]	-0.55 (5.37)	[-11.41, 10.40]	10.79 (8.75)	[-6.88, 28.75]	-12.38 (13.93)	[-40.61, 15.57]
Prop. of fibroblasts	-26.39 (8.76)*	[-43.63, -9.24]	-6.31 (7.28)	[-20.72, 7.91]	-15.12 (12.45)	[-39.79, 9.03]	40.13 (20.19)	[-0.54, 79.32]
Prop. of immune cells	-0.48 (7.31)	[-14.76, 13.79]	0.45 (5.37)	[-10.36, 11.40]	-11.02 (8.75)	[-28.67, 6.92]	12.73 (13.92)	[-15.67, 40.80]
City 2 (vs. City 1)	0.03 (0.28)	[-0.51, 0.58]	-0.02 (0.28)	[-0.55, 0.53]	-0.17 (0.51)	[-1.18, 0.82]	0.60 (0.82)	[-1.04, 2.19]
City 3 (vs. City 1)	-0.23 (0.29)	[-0.79, 0.36]	-0.72 (0.29)	[-1.29, -0.14]	-0.76 (0.53)	[-1.79, 0.28]	0.07 (0.85)	[-1.58, 1.70]
City 4 (vs. City 1)	-0.01 (0.28)	[-0.57, 0.53]	-0.76 (0.27)	[-1.30, -0.21]	-0.75 (0.50)	[-1.73, 0.23]	-0.12 (0.81)	[-1.71, 1.46]
City 5 (vs. City 1)	0.28 (0.29)	[-0.27, 0.84]	-0.34 (0.29)	[-0.90, 0.22]	-0.39 (0.52)	[-1.41, 0.62]	0.03 (0.85)	[-1.66, 1.69]

© 2024 Del Toro J et al. *JAMA Pediatrics.*

City 6 (vs. City 1)	-0.02 (0.28)	[-0.58, 0.52]	-0.67 (0.28)	[-1.22, -0.11]	-0.52 (0.52)	[-1.55, 0.49]	-0.40 (0.84)	[-2.06, 1.22]
City 7 (vs. City 1)	-0.02 (0.28)	[-0.56, 0.52]	-0.91 (0.28)*	[-1.47, -0.37]	-0.43 (0.51)	[-1.42, 0.57]	-0.27 (0.83)	[-1.91, 1.33]
City 8 (vs. City 1)	-0.11 (0.27)	[-0.64, 0.42]	-0.81 (0.27)*	[-1.33, -0.27]	-0.25 (0.49)	[-1.22, 0.71]	-0.92 (0.78)	[-2.42, 0.63]
City 9 (vs. City 1)	0.19 (0.27)	[-0.34, 0.72]	-0.84 (0.27)*	[-1.38, -0.33]	-0.85 (0.49)	[-1.80, 0.10]	-0.85 (0.78)	[-2.38, 0.69]
City 10 (vs. City 1)	-0.26 (0.27)	[-0.79, 0.26]	-1.32 (0.27)*	[-1.85, -0.78]	-0.49 (0.49)	[-1.45, 0.46]	-0.41 (0.79)	[-1.96, 1.13]
City 11 (vs. City 1)	-0.62 (0.31)	[-1.22, -0.01]	-1.29 (0.31)*	[-1.90, -0.70]	-1.82 (0.56)*	[-2.91, -0.72]	-1.83 (0.91)	[-3.63, -0.07]
City 12 (vs. City 1)	-0.07 (0.29)	[-0.63, 0.49]	-0.86 (0.28)*	[-1.41, -0.30]	-0.89 (0.52)	[-1.92, 0.11]	-1.54 (0.84)	[-3.20, 0.09]
City 13 (vs. City 1)	-0.35 (0.40)	[-0.44, 1.12]	-0.87 (0.39)	[-1.63, -0.11]	0.08 (0.74)	[-1.34, 1.53]	0.27 (1.17)	[-2.02, 2.58]
City 14 (vs. City 1)	-0.13 (0.37)	[-0.85, 0.60]	-1.17 (0.37)*	[-1.90, -0.47]	-0.76 (0.69)	[-2.11, 0.60]	-1.83 (1.11)	[-4.01, 0.38]
City 15 (vs. City 1)	0.07 (0.36)	[-0.64, 0.79]	-1.10 (0.36)*	[-1.81, -0.39]	-0.63 (0.68)	[-2.00, 0.68]	-0.82 (1.10)	[-3.00, 1.33]
City 16 (vs. City 1)	-0.54 (0.40)	[-1.34, 0.25]	-1.53 (0.40)*	[-2.31, -0.76]	-1.45 (0.73)	[-2.89, -0.04]	-0.91 (1.17)	[-3.21, 1.42]
City 17 (vs. City 1)	0.15 (0.38)	[-0.59, 0.89]	-1.08 (0.38)*	[-1.83, -0.35]	-0.80 (0.69)	[-2.17, 0.56]	-1.66 (1.11)	[-3.85, 0.49]
City 18 (vs. City 1)	-0.20 (0.36)	[-0.91, 0.51]	-1.34 (0.36)*	[-2.05, -0.62]	0.09 (0.65)	[-1.18, 1.37]	-1.23 (1.06)	[-3.33, 0.83]
City 19 (vs. City 1)	0.13 (0.37)	[-0.58, 0.87]	-0.94 (0.38)*	[-1.67, -0.22]	-0.35 (0.68)	[-1.68, 0.97]	-1.07 (1.09)	[-3.21, 1.08]
City 20 (vs. City 1)	0.05 (0.37)	[-0.68, 0.78]	-1.02 (0.37)*	[-1.76, -0.30]	-0.39 (0.69)	[-1.74, 0.97]	-1.47 (1.09)	[-3.65, 0.66]
Intercepts	0.00 (0.05)	[-0.08, 0.10]	0.04 (0.05)	[-0.05, 0.13]	0.00 (0.09)	[-0.16, 0.18]	-0.09 (0.14)	[-0.37, 0.18]
Random effects								
Outcome – variance	3.90 (0.23)*	[3.46, 4.31]	2.81 (0.73)*	[1.55, 3.88]	13.25 (1.01)*	[10.97, 14.77]	32.70 (3.64)*	[24.63, 37.79]
Covariance – intercept and slope	2.43 (0.11)*	[2.21, 2.66]	--	--	15.66 (0.64)*	[14.44, 16.99]	--	--

Note: * *Bayesian significance*, after we used the Benjamini-Hochberg FDR method to account for multiple testing.

© 2024 Del Toro J et al. *JAMA Pediatrics.*

eTable 6

Posterior Estimates (SD) from a Bayesian Multilevel Model Examining Changes in DNA Methylation-Age among 2,039 Youth Transitioning Between Childhood and Adolescence

	GrimAge				PhenoAge			
	Intercept		Slope		Intercept		Slope	
Measures	*Estimate (SD)*	*95% CI*	*Estimate (SD)*	*95% CI*	*Estimate (SD)*	*95% CI*	*Estimate (SD)*	*95% CI*
Fixed effects								
Black (vs. White)	0.31 (0.20)	[-0.07, 0.72]	1.36 (0.33)*	[0.73, 2.00]	-0.39 (0.37)	[-1.12, 0.34]	0.46 (0.41)	[-0.31, 1.30]
Other (vs. White)	0.38 (0.22)	[-0.05, 0.83]	0.71 (0.35)*	[0.02, 1.40]	-0.05 (0.40)	[-0.84, 0.71]	0.10 (0.45)	[-0.77, 0.98]
Latinx (vs. White)	0.52 (0.24)	[0.06, 1.02]	0.54 (0.38)	[-0.16, 1.29]	0.62 (0.45)	[-0.26, 1.53]	0.22 (0.49)	[-0.74, 1.18]
Female (vs. male)	-0.85 (0.12)*	[-1.08, -0.61]	0.40 (0.18)*	[0.05, 0.75]	2.05 (0.22)*	[1.61, 2.48]	0.19 (0.25)	[-0.29, 0.68]
Child's age	0.39 (0.17)	[0.04, 0.73]	-0.62 (0.26)*	[-1.13, -0.10]	0.93 (0.32)*	[0.29, 1.55]	-1.42 (0.36)*	[-2.15, -0.71]
Family SES	-0.24 (0.08)*	[-0.39, -0.09]	-0.10 (0.11)	[-0.31, 0.13]	-0.34 (0.14)*	[-0.62, -0.08]	0.04 (0.16)	[-0.27, 0.35]
Child smoking	0.03 (0.61)	[-1.13, 1.23]	0.90 (0.91)	[-0.91, 2.64]	-0.35 (1.12)	[-2.57, 1.84]	-0.34 (1.28)	[-2.88, 2.16]
Maternal smoking	-0.11 (0.16)	[-0.43, 0.20]	0.25 (0.23)	[-0.19, 0.69]	0.17 (0.28)	[-0.38, 0.73]	0.36 (0.31)	[-0.26, 0.98]
DNAm smoking	1.18 (0.06)*	[1.05, 1.30]	-0.63 (0.09)*	[-0.82, -0.45]	0.81 (0.11)*	[0.58, 1.03]	-0.56 (0.13)*	[-0.83, -0.31]
Child BMI	0.27 (0.05)*	[0.16, 0.38]	0.05 (0.08)	[-0.11, 0.21]	0.45 (0.10)*	[0.26, 0.64]	0.06 (0.12)	[-0.16, 0.28]
Child puberty	0.11 (0.11)	[-0.10, 0.33]	-0.18 (0.16)	[-0.50, 0.15]	0.04 (0.21)	[-0.37, 0.43]	-0.11 (0.23)	[-0.57, 0.35]
EPIC (vs. 450K)	0.00 (0.13)	[-0.25, 0.25]	-0.54 (0.20)*	[-0.93, -0.15]	-0.11 (0.24)	[-0.60, 0.35]	-0.29 (0.27)	[-0.84, 0.21]
Prop. of epithelial cells	6.96 (3.95)	[-1.83, 15.52]	-7.44 (5.56)	[-19.63, 4.71]	5.10 (15.31)	[-25.66, 34.57]	-4.74 (12.62)	[-31.28, 21.45]
Prop. of fibroblasts	-12.86 (7.18)	[-26.95, 1.32]	39.91 (10.87)*	[19.13, 61.43]	-100.95 (18.78)*	[-139.43, -65.61]	90.35 (17.57)*	[56.15, 126.97]
Prop. of immune cells	-6.99 (3.96)	[-15.86, 1.46]	7.54 (5.57)	[-4.67, 19.68]	-3.69 (15.30)	[-34.52, 25.60]	4.59 (12.64)	[-22.10, 30.69]
City 2 (vs. City 1)	0.02 (0.33)	[-0.64, 0.65]	-0.03 (0.56)	[-1.16, 1.03]	0.44 (0.63)	[-0.79, 1.67]	-0.13 (0.71)	[-1.51, 1.25]
City 3 (vs. City 1)	0.14 (0.34)	[-0.53, 0.78]	-0.48 (0.57)	[-1.63, 0.58]	-0.41 (0.65)	[-1.73, 0.84]	-0.71 (0.78)	[-2.23, 0.89]
City 4 (vs. City 1)	0.13 (0.33)	[-0.52, 0.75]	-0.30 (0.54)	[-1.40, 0.67]	-0.24 (0.62)	[-1.45, 0.98]	-0.59 (0.72)	[-1.97, 0.83]
City 5 (vs. City 1)	0.11 (0.34)	[-0.56, 0.77]	-0.24 (0.57)	[-1.42, 0.79]	-0.42 (0.64)	[-1.66, 0.83]	0.00 (0.74)	[-1.49, 1.39]

© 2024 Del Toro J et al. *JAMA Pediatrics.*

City 6 (vs. City 1)	0.02 (0.34)	[-0.68, 0.65]	-0.50 (0.54)	[-1.61, 0.46]	-0.28 (0.64)	[-1.55, 0.99]	-1.34 (0.71)	[-2.76, 0.01]
City 7 (vs. City 1)	-0.48 (0.33)	[-1.15, 0.14]	-0.54 (0.54)	[-1.66, 0.41]	0.15 (0.63)	[-1.10, 1.36]	-1.61 (0.71)*	[-2.99, -0.26]
City 8 (vs. City 1)	-0.34 (0.32)	[-1.01, 0.26]	-1.28 (0.54)*	[-2.37, -0.32]	-0.08 (0.60)	[-1.29, 1.11]	-1.27 (0.68)	[-2.62, 0.02]
City 9 (vs. City 1)	-0.18 (0.31)	[-0.83, 0.39]	-1.07 (0.51)*	[-2.14, -0.17]	-1.31 (0.60)	[-2.48, -0.17]	-1.44 (0.67)	[-2.76, -0.11]
City 10 (vs. City 1)	0.08 (0.32)	[-0.58, 0.66]	-0.65 (0.52)	[-1.72, 0.27]	-0.40 (0.60)	[-1.60, 0.76]	-1.50 (0.72)	[-2.86, -0.07]
City 11 (vs. City 1)	-0.35 (0.38)	[-1.10, 0.35]	-1.72 (0.63)*	[-2.98, -0.54]	-2.78 (0.69)*	[-4.15, -1.44]	-2.22 (0.80)*	[-3.81, -0.68]
City 12 (vs. City 1)	-0.24 (0.35)	[-0.96, 0.41]	-1.49 (0.57)*	[-2.63, -0.41]	-0.32 (0.64)	[-1.57, 0.95]	-2.00 (0.72)*	[-3.38, -0.55]
City 13 (vs. City 1)	-0.30 (0.47)	[-1.26, 0.60]	-1.16 (0.76)	[-2.72, 0.30]	-0.73 (0.90)	[-2.49, 1.04]	-1.61 (1.00)	[-3.52, 0.36]
City 14 (vs. City 1)	-0.66 (0.45)	[-1.60, 0.19]	-1.58 (0.70*	[-3.00, -0.28]	-1.72 (0.85)	[-3.43, -0.06]	-2.52 (0.98)*	[-4.46, -0.63]
City 15 (vs. City 1)	0.28 (0.43)	[-0.61, 1.09]	-1.04 (0.72)	[-2.54, 0.25]	-0.49 (0.83)	[-2.15, 1.09]	-1.29 (0.93)	[-3.52, 0.36]
City 16 (vs. City 1)	-0.42 (0.49)	[-1.39, 0.53]	-1.77 (0.76)*	[-3.29, -0.33]	-2.89 (0.90)*	[-4.63, -1.06]	-2.47 (1.03)*	[-4.47, -0.44]
City 17 (vs. City 1)	-0.49 (0.46)	[-1.41, 0.39]	-1.43 (0.72)*	[-2.91, -0.11]	-1.03 (0.86)	[-2.70, 0.67]	-1.27 (0.96)	[-3.15, 0.59]
City 18 (vs. City 1)	-0.56 (0.45)	[-1.47, 0.29]	-1.50 (0.71)*	[-2.95, -0.20]	-0.31 (0.82)	[-1.90, 1.31]	-1.55 (0.91)	[-3.51, 0.17]
City 19 (vs. City 1)	-0.08 (0.44)	[-0.97, 0.79]	-1.07 (0.68)	[-2.49, 0.21]	-0.23 (0.84)	[-1.88, 1.40]	-2.13 (0.98)*	[-4.08, -0.26]
City 20 (vs. City 1)	-0.11 (0.45)	[-1.01, 0.76]	-1.28 (0.72)	[-2.74, 0.11]	-1.29 (0.83)	[-2.94, 0.34]	-2.36 (0.95)*	[-4.19, -0.44]
Intercepts	0.03 (0.06)	[-0.09, 0.14]	-0.12 (0.09)	[-0.29, 0.05]	-0.01 (0.10)	[-0.22, 0.19]	0.07 (0.12)	[-0.18, 0.30]
Random effects								
Variance – outcome	5.30 (0.50)*	[4.75, 6.61]	9.08 (1.83)*	[7.63, 14.25]	17.92 (1.74)*	[14.62, 21.07]	11.83 (6.14)*	[1.21, 22.28]
Covariance – intercept and slope	6.40 (0.28)*	[5.86, 6.96]	--	--	3.01 (0.56)*	[1.91, 4.10]	--	--

Note: * *Bayesian significance*, after we used the Benjamini-Hochberg FDR method to account for multiple testing.

© 2024 Del Toro J et al. *JAMA Pediatrics.*

eTable 7

Posterior Estimates (SD) from a Bayesian Multilevel Model Examining Changes in Epigenetic Age Acceleration among 2,039 Youth Transitioning Between Childhood and Adolescence

	Horvath				Hannum			
	Intercept		Slope		Intercept		Slope	
Measures	*Estimate (SD)*	*95% CI*	*Estimate (SD)*	*95% CI*	*Estimate (SD)*	*95% CI*	*Estimate (SD)*	*95% CI*
Fixed effects								
Black (vs. White)	-0.52 (0.16)*	[-0.83, -0.21]	-0.24 (0.16)	[-0.54, 0.05]	-0.80 (0.30)*	[-1.38, -0.21]	1.54 (0.49)*	[0.36, 2.18]
Other (vs. White)	-0.14 (0.17)	[-0.48, 0.20]	-0.15 (0.17)	[-0.48, 0.18]	-0.37 (0.32)	[-1.00, 0.25]	0.71 (0.53)	[-0.55, 1.44]
Latinx (vs. White)	-0.10 (0.19)	[-0.49, 0.27]	-0.18 (0.18)	[-0.54, 0.17]	0.05 (0.36)	[-0.66, 0.76]	0.90 (0.59)	[-0.42, 1.71]
Female (vs. male)	0.07 (0.09)	[-0.12, 0.25]	-0.13 (0.09)	[-0.31, 0.05]	-0.61 (0.18)*	[-0.95, -0.26]	-0.55 (0.28)	[-1.15, -0.06]
Child age	0.13 (0.14)	[-0.14, 0.40]	-0.26 (0.14)	[-0.53, 0.01]	0.29 (0.26)	[-0.22, 0.80]	-0.10 (0.42)	[-0.84, 0.68]
Family SES	0.06 (0.06)	[-0.06, 0.18]	-0.04 (0.06)	[-0.16, 0.08]	-0.03 (0.12)	[-0.21, 0.20]	0.05 (0.19)	[-0.35, 0.36]
Child smoking	0.17 (0.48)	[-0.78, 1.11]	0.51 (0.47)	[-0.43, 1.42]	0.46 (0.91)	[-1.31, 2.25]	2.28 (1.50)	[-1.04, 5.26]
Maternal smoking	-0.19 (0.13)	[-0.44, 0.06]	0.10 (0.12)	[-0.14, 0.33]	-0.01 (0.23)	[-0.47, 0.44]	0.29 (0.38)	[-0.47, 0.96]
DNAm smoking	0.50 (0.05)*	[0.40, 0.60]	0.11 (0.05)	[0.01, 0.21]	-0.12 (0.09)	[-0.30, 0.07]	-0.56 (0.15)*	[-0.79, -0.19]
Child BMI	0.16 (0.04)*	[0.07, 0.24]	0.11 (0.04)	[0.02, 0.19]	0.22 (0.08)*	[0.06, 0.38]	-0.05 (0.13)	[-0.30, 0.19]
Child puberty	-0.10 (0.09)	[-0.27, 0.08]	-0.06 (0.08)	[-0.22, 0.11]	0.06 (0.16)	[-0.26, 0.38]	-0.04 (0.27)	[-0.59, 0.51]
EPIC (vs. 450K)	0.09 (0.10)	[-0.11, 0.30]	0.03 (0.10)	[-0.18, 0.22]	0.28 (0.19)	[-0.11, 0.67]	-0.33 (0.32)	[-0.84, 0.31]
Prop. of epithelial cells	0.62 (4.56)	[-9.11, 10.08]	-0.47 (3.99)	[-9.24, 7.96]	10.81 (8.72)	[-6.85, 28.76]	-12.39 (13.87)	[-25.90, 1.51]
Prop. of fibroblasts	-24.80 (6.61)*	[-37.98, -11.80]	-3.94 (6.21)	[-15.69, 8.67]	-13.97 (12.43)	[-38.66, 10.17]	42.71 (20.16)	[14.73, 17.50]
Prop. of immune cells	-0.24 (4.57)	[-10.03, 9.34]	0.49 (3.99)	[-8.27, 8.95]	-11.03 (8.72)	[-28.66, 6.90]	12.73 (13.86)	[-1.34, 26.16]
City 2 (vs. City 1)	0.05 (0.27)	[-0.48, 0.58]	0.04 (0.27)	[-0.49, 0.56]	-0.15 (0.51)	[-1.17, 0.81]	0.55 (0.82)	[-0.76, 2.04]
City 3 (vs. City 1)	0.04 (0.27)	[-0.47, 0.58]	-0.20 (0.28)	[-0.75, 0.35]	-0.48 (0.53)	[-1.50, 0.56]	0.56 (0.85)	[-0.89, 2.11]

© 2024 Del Toro J et al. *JAMA Pediatrics.*

City 4 (vs. City 1)	0.19 (0.27)	[-0.31, 0.73]	-0.38 (0.27)	[-0.91, 0.14]	-0.54 (0.50)	[-1.53, 0.44]	0.22 (0.81)	[-1.32, 1.48]
City 5 (vs. City 1)	0.45 (0.27)	[-0.06, 1.00]	-0.03 (0.28)	[-0.58, 0.51]	-0.20 (0.51)	[-1.23, 0.81]	0.31 (0.85)	[-0.95, 1.92]
City 6 (vs. City 1)	0.28 (0.27)	[-0.25, 0.82]	-0.09 (0.28)	[-0.64, 0.45]	-0.21 (0.52)	[-1.24, 0.80]	0.14 (0.84)	[-1.24, 1.62]
City 7 (vs. City 1)	0.31 (0.26)	[-0.21, 0.84]	-0.25 (0.27)	[-0.79, 0.27]	-0.08 (0.51)	[-1.07, 0.91]	0.37 (0.83)	[-0.86, 1.87]
City 8 (vs. City 1)	0.28 (0.25)	[-0.23, 0.78]	-0.03 (0.26)	[-0.54, 0.47]	0.16 (0.49)	[-0.81, 1.12]	-0.16 (0.78)	[-1.26, 1.42]
City 9 (vs. City 1)	0.59 (0.25)	[0.12, 1.07]	-0.07 (0.26)	[-0.57, 0.43]	-0.43 (0.49)	[-1.37, 0.52]	-0.09 (0.78)	[-1.37, 1.24]
City 10 (vs. City 1)	0.15 (0.25)	[-0.35 0.64]	-0.52 (0.26)	[-1.02, -0.01]	-0.08 (0.49)	[-1.03, 0.88]	0.38 (0.79)	[-0.96, 1.65]
City 11 (vs. City 1)	-0.16 (0.29)	[-0.73, 0.40]	-0.42 (0.30)	[-1.01, 0.16]	-1.34 (0.56)	[-2.44, -0.25]	-0.96 (0.91)	[-2.45, 0.63]
City 12 (vs. City 1)	0.40 (0.27)	[-0.12, 0.94]	0.09 (0.27)	[-0.45, 0.62]	-0.40 (0.52)	[-1.42, 0.61]	-0.60 (0.84)	[-1.80, 1.06]
City 13 (vs. City 1)	0.73 (0.38)	[-0.01, 1.47]	-0.11 (0.38)	[-0.87, 0.64]	0.49 (0.73)	[-0.92, 1.93]	1.01 (1.17)	[-1.17, 3.05]
City 14 (vs. City 1)	0.29 (0.37)	[-0.43, 0.98]	-0.40 (0.36)	[-1.11, 0.30]	-0.34 (0.69)	[-1.69, 1.02]	-1.07 (1.11)	[-2.83, 1.05]
City 15 (vs. City 1)	0.43 (0.35)	[-0.23, 1.12]	-0.41 (0.36)	[-1.11, 0.29]	-0.26 (0.68)	[-1.63, 1.05]	-0.16 (1.10)	[-1.95, 2.04]
City 16 (vs. City 1)	-0.09 (0.40)	[-0.83, 0.69]	-0.67 (0.39)	[-1.43, 0.09]	-0.98 (0.73)	[-2.42, 0.43]	-0.06 (1.17)	[-2.38, 1.84]
City 17 (vs. City 1)	0.49 (0.36)	[-0.20, 1.21]	-0.44 (0.36)	[-1.15, 0.26]	-0.45 (0.69)	[-1.82, 0.92]	-1.04 (1.11)	[-2.98, 1.07]
City 18 (vs. City 1)	0.26 (0.35)	[-0.43, 0.93]	-0.40 (0.35)	[-1.09, 0.27]	0.58 (0.65)	[-0.69, 1.86]	-0.28 (1.06)	[-2.00, 1.07]
City 19 (vs. City 1)	0.54 (0.35)	[-0.15, 1.25]	-0.17 (0.36)	[-0.86, 0.54]	0.06 (0.68)	[-1.27, 1.38]	-0.30 (1.10)	[-2.27, 1.57]
City 20 (vs. City 1)	0.54 (0.35)	[-0.15, 1.22]	-0.09 (0.36)	[-0.78, 0.61]	0.11 (0.68)	[-1.23, 1.47]	-0.56 (1.09)	[-2.34, 1.51]
Intercepts	0.01 (0.05)	[-0.08, 0.10]	0.05 (0.05)	[-0.04, 0.14]	0.01 (0.09)	[-0.16, 0.18]	-0.08 (0.14)	[-0.36, 0.19]
Random effects								
Variance – outcome	3.83 (0.22)*	[3.41, 4.25]	2.54 (0.70)*	[1.43, 3.68]	5.84 (0.81)*	[4.39, 7.57]	2.52 (2.10)*	[0.21, 7.98]
Covariance – intercept and slope	2.33 (0.11)*	[2.12, 2.56]	--	--	15.61 (0.64)*	[14.39, 16.93]	--	--

Note: * *Bayesian significance*, after we used the Benjamini-Hochberg FDR method to account for multiple testing.

© 2024 Del Toro J et al. *JAMA Pediatrics.*

eTable 8

Posterior Estimates (SD) from a Bayesian Multilevel Model Examining Changes in Epigenetic Age Acceleration among 2,039 Youth Transitioning Between Childhood and Adolescence

	GrimAge				PhenoAge			
	Intercept		Slope		Intercept		Slope	
Measures	*Estimate (SD)*	*95% CI*	*Estimate (SD)*	*95% CI*	*Estimate (SD)*	*95% CI*	*Estimate (SD)*	*95% CI*
Fixed effects								
Black (vs. White)	0.29 (0.20)	[-0.08, 0.70]	1.31 (0.33)*	[0.68, 1.97]	-0.41 (0.37)	[-1.12, 0.32]	0.46 (0.41)	[-0.34, 1.29]
Other (vs. White)	0.36 (0.21)	[-0.06, 0.80]	0.67 (0.35)	[-0.03, 1.35]	-0.09 (0.39)	[-0.85, 0.69]	0.10 (0.44)	[-0.74, 0.97]
Latinx (vs. White)	0.49 (0.25)	[0.04, 1.00]	0.49 (0.38)	[-0.21, 1.24]	0.61 (0.45)	[-0.27, 1.48]	0.19 (0.50)	[-0.76, 1.19]
Female (vs. male)	-0.83 (0.12)*	[-1.07, -0.60]	0.44 (0.18)	[0.09, 0.78]	2.06 (0.22)*	[1.63, 2.48]	0.25 (0.24)	[-0.21, 0.73]
Child age	0.11 (0.17)	[-0.23, 0.44]	-0.52 (0.25)	[-1.01, -0.02]	0.01 (0.32)	[-0.62, 0.65]	0.00 (0.35)	[-0.70, 0.69]
Family SES	-0.22 (0.08)*	[-0.37, -0.06]	-0.05 (0.11)	[-0.26, 0.18]	-0.31 (0.14)	[-0.58, -0.03]	0.10 (0.16)	[-0.21, 0.41]
Child smoking	0.05 (0.61)	[-1.11, 1.25]	0.94 (0.90)	[-0.86, 2.66]	-0.31 (1.12)	[-2.51, 1.86]	-0.28 (1.27)	[-2.82, 2.14]
Maternal smoking	-0.13 (0.16)	[-0.44, 0.19]	0.23 (0.23)	[-0.21,0.67]	0.14 (0.28)	[-0.42, 0.71]	0.34 (0.32)	[-0.29, 0.94]
DNAm smoking	1.17 (0.06)*	[1.04, 1.29]	-0.64 (0.09)*	[-0.83, -0.47]	0.80 (0.11)*	[0.57, 1.03]	-0.58 (0.13)*	[-0.84, -0.33]
Child BMI	0.27 (0.05)*	[0.16, 0.37]	0.04 (0.08)	[-0.12, 0.21]	0.45 (0.10)*	[0.25, 0.64]	0.06 (0.11)	[-0.16, 0.28]
Child puberty	0.12 (0.11)	[-0.10, 0.33]	-0.17 (0.16)	[-0.50, 0.14]	0.05 (0.21)	[-0.36, 0.44]	-0.10 (0.23)	[-0.56, 0.35]
EPIC (vs. 450K)	0.10 (0.13)	[-0.15, 0.35]	-0.32 (0.19)	[-0.70, 0.06]	0.01 (0.24)	[-0.46, 0.47]	0.01 (0.26)	[-0.53, 0.52]
Prop. of epithelial cells	6.94 (3.75)	[-1.28, 15.11]	-7.42 (5.26)	[-18.69, 4.24]	4.96 (15.56)	[-21.63, 31.08]	-4.62 (12.38)	[-30.49, 20.09]
Prop. of fibroblasts	-11.28 (7.06)	[-25.01, 2.83]	43.26 (10.70)*	[23.02, 64.84]	-99.03 (18.92)*	[-133.90, -65.23]	94.90 (17.47)*	[61.07, 130.46]
Prop. of immune cells	-6.99 (3.76)	[-15.43, 1.01]	7.54 (5.27)	[-3.75, 19.19]	-3.84 (15.56)	[-30.39, 22.25]	4.67 (12.39)	[-21.34, 29.60]
City 2 (vs. City 1)	-0.11 (0.33)	[-0.74, 0.55]	-0.24 (0.50)	[-1.27, 0.72]	0.50 (0.62)	[-0.71, 1.73]	-0.21 (0.71)	[-1.60, 1.18]

© 2024 Del Toro J et al. *JAMA Pediatrics.*

City 3 (vs. City 1)	0.38 (0.33)	[-0.24, 1.04]	0.04 (0.50)	[-0.93, 1.03]	0.16 (0.65)	[-1.11, 1.44]	0.19 (0.75)	[-1.30, 1.67]
City 4 (vs. City 1)	0.26 (0.32)	[-0.32, 0.93]	-0.01 (0.46)	[-0.92, 0.89]	0.18 (0.62)	[-1.02, 1.40]	0.01 (0.69)	[-1.32, 1.35]
City 5 (vs. City 1)	0.21 (0.33)	[-0.41, 0.86]	-0.01 (0.49)	[-0.99, 0.91]	-0.05 (0.64)	[-1.31, 1.21]	0.45 (0.72)	[-1.00, 1.82]
City 6 (vs. City 1)	0.30 (0.33)	[-0.33, 0.94]	0.09 (0.47)	[-0.84, 1.05]	0.32 (0.64)	[-0.92, 1.60]	-0.35 (0.71)	[-1.75, 1.05]
City 7 (vs. City 1)	-0.14 (0.31)	[-0.73, 0.50]	0.18 (0.47)	[-0.73, 1.11]	0.81 (0.63)	[-0.39, 2.05]	-0.44 (0.71)	[-1.79, 0.92]
City 8 (vs. City 1)	0.09 (0.30)	[-0.49, 0.69]	-0.35 (0.45)	[-1.27, 0.50]	0.70 (0.60)	[-0.47, 1.90]	0.10 (0.66)	[-1.19, 1.40]
City 9 (vs. City 1)	0.26 (0.30)	[-0.31, 0.85]	-0.17 (0.44)	[-1.03, 0.71]	-0.51 (0.60)	[-1.65, 0.69]	-0.07 (0.67)	[-1.35, 1.27]
City 10 (vs. City 1)	0.52 (0.30)	[-0.07, 1.13]	0.30 (0.44)	[-0.57, 1.17]	0.40 (0.60)	[-0.78, 1.60]	-0.06 (0.69)	[-1.37, 1.27]
City 11 (vs. City 1)	0.18 (0.35)	[-0.52, 0.85]	-0.62 (0.53)	[-1.70, 0.42]	-1.87 (0.69)*	[-3.22, -0.52]	-0.66 (0.77)	[-1.17, 0.86]
City 12 (vs. City 1)	0.32 (0.32)	[-0.30, 0.95]	-0.31 (0.48)	[-1.25, 0.62]	0.62 (0.65)	[-0.62, 1.86]	-0.25 (0.72)	[-1.65, 1.18]
City 13 (vs. City 1)	0.12 (0.46)	[-0.78, 1.01]	-0.29 (0.68)	[-1.65, 1.05]	0.06 (0.89)	[-1.69, 1.83]	-0.22 (0.99)	[-2.15, 1.75]
City 14 (vs. City 1)	-0.22 (0.43)	[-1.10, 0.63]	-0.68 (0.64)	[-1.93, 0.58]	-0.92 (0.85)	[-2.56, 0.74]	-1.15 (0.96)	[-3.04, 0.71]
City 15 (vs. City 1)	0.64 (0.43)	[-0.16, 1.50]	-0.28 (0.64)	[-1.56, 0.98]	0.20 (0.84)	[-1.42, 1.82]	-0.08 (0.94)	[-1.90, 1.77]
City 16 (vs. City 1)	0.09 (0.46)	[-0.82, 1.03]	-0.71 (0.71)	[-2.09, 0.71]	-1.97 (0.90)	[-3.76, -0.22]	-0.94 (1.01)	[-2.91, 1.06]
City 17 (vs. City 1)	-0.15 (0.44)	[-1.00, 0.72]	-0.71 (0.66)	[-2.04, 0.58]	-0.35 (0.85)	[-1.99, 1.33]	-0.11 (0.94)	[-1.97, 1.75]
City 18 (vs. City 1)	0.00 (0.42)	[-0.84, 0.82]	-0.33 (0.63)	[-1.61, 0.87]	0.61 (0.82)	[-0.97, 2.19]	0.17 (0.89)	[-1.64, 1.88]
City 19 (vs. City 1)	0.34 (0.43)	[-0.49, 1.23]	-0.18 (0.64)	[-1.41, 1.08]	0.57 (0.84)	[-1.09, 2.17]	-0.77 (0.96)	[-2.69, 1.09]
City 20 (vs. City 1)	0.45 (0.43)	[-0.38, 1.29]	-0.14 (0.66)	[-1.43, 1.15]	-0.32 (0.83)	[-1.95, 1.32]	-0.69 (0.93)	[-2.50, 1.12]
Intercepts	0.04 (0.06)	[-0.08, 0.15]	-0.10 (0.08)	[-0.27, 0.06]	0.00 (0.11)	[-0.21, 0.21]	0.09 (0.12)	[-0.14, 0.32]
Random effects								
Variance – outcome	5.21 (0.46)*	[4.68, 6.49]	8.86 (1.70)*	[7.48, 13.91]	17.67 (1.59)*	[14.92, 20.91]	10.65 (5.46)	[2.33, 21.63]
Covariance – intercept and slope	6.30 (0.28)*	[5.78, 6.87]	--	--	2.79 (0.57)	[1.65, 3.88]	--	--

Note: * *Bayesian significance,* after we used the Benjamini-Hochberg FDR method to account for multiple testing.

© 2024 Del Toro J et al. *JAMA Pediatrics.*

eTable 9

Posterior Estimates (SD) from a Bayesian Multilevel Model Examining Changes in Epigenetic Age Acceleration among 2,039 Youth Transitioning Between Childhood and Adolescence

	DunedinPACE			
	Intercept		Slope	
Measures	*Estimate (SD)*	*95% CI*	*Estimate (SD)*	*95% CI*
Fixed effects				
Black (vs. White)	0.27 (0.05)*	[0.17, 0.37]	0.27 (0.09)*	[0.11, 0.44]
Other (vs. White)	0.13 (0.05)*	[0.02, 0.23]	0.14 (0.09)	[-0.04, 0.31]
Latinx (vs. White)	0.22 (0.06)*	[0.10, 0.35]	0.12 (0.10)	[-0.05, 0.32]
Female (vs. male)	0.12 (0.03)*	[0.07, 0.18]	0.21 (0.05)*	[0.12, 0.30]
Child age	-0.03 (0.04)	[-0.12, 0.05]	-0.04 (0.07)	[-0.17, 0.09]
Family SES	-0.09 (0.02)*	[-0.13, -0.05]	0.03 (0.03)	[-0.03, 0.09]
Child smoking	0.18 (0.15)	[-0.10, 0.47]	0.41 (0.23)	[-0.05, 0.86]
Maternal smoking	0.00 (0.04)	[-0.08, 0.07]	0.10 (0.06)	[-0.01, 0.21]
DNAm smoking	-0.02 (0.02)	[-0.05, 0.01]	-0.08 (0.02)*	[-0.13, -0.04]
Child BMI	0.12 (0.01)*	[0.10, 0.15]	0.00 (0.02)	[-0.04, 0.05]
Child puberty	-0.03 (0.03)	[-0.08, 0.02]	-0.01 (0.04)	[-0.09, 0.08]
EPIC (vs. 450K)	0.00 (0.03)	[-0.06, 0.06]	-0.10 (0.05)	[-0.20, -0.01]
Prop. of epithelial cells	1.80 (1.28)	[-0.97, 4.50]	-2.25 (1.86)	[-6.39, 1.75]
Prop. of fibroblasts	5.28 (1.92)*	[1.33, 8.95]	-1.18 (2.99)	[-6.93, 4.80]
Prop. of immune cells	-1.84 (1.28)	[-4.66, 0.86]	2.33 (1.86)	[-1.80, 6.40]
City 2 (vs. City 1)	0.08 (0.08)	[-0.07, 0.24]	-0.04 (0.14)	[-0.32, 0.22]
City 3 (vs. City 1)	-0.06 (0.09)	[-0.22, 0.10]	0.08 (0.13)	[-0.18, 0.34]
City 4 (vs. City 1)	-0.02 (0.08)	[-0.17, 0.14]	0.06 (0.12)	[-0.19, 0.29]

© 2024 Del Toro J et al. *JAMA Pediatrics.*

City 5 (vs. City 1)	0.00 (0.09)	[-0.16, 0.16]	-0.01 (0.13)	[-0.28, 0.23]
City 6 (vs. City 1)	0.04 (0.08)	[-0.12, 0.20]	-0.02 (0.12)	[-0.27, 0.22]
City 7 (vs. City 1)	-0.05 (0.08)	[-0.21, 0.10]	0.03 (0.12)	[-0.22, 0.27]
City 8 (vs. City 1)	-0.08 (0.08)	[-0.23, 0.07]	-0.09 (0.12)	[-0.34, 0.13]
City 9 (vs. City 1)	-0.10 (0.08)	[-0.25, 0.04]	-0.11 (0.12)	[-0.35, 0.11]
City 10 (vs. City 1)	0.08 (0.08)	[-0.07, 0.22]	0.03 (0.12)	[-0.22, 0.25]
City 11 (vs. City 1)	-0.07 (0.09)	[-0.25, 0.09]	-0.31 (0.14)	[-0.61, -0.04]
City 12 (vs. City 1)	-0.07 (0.08)	[-0.24, 0.09]	-0.13 (0.13)	[-0.39, 0.12]
City 13 (vs. City 1)	0.05 (0.11)	[-0.17, 0.27]	-0.18 (0.18)	[-0.54, 0.17]
City 14 (vs. City 1)	0.02 (0.11)	[-0.21, 0.21]	-0.33 (0.17)	[-0.65, -0.01]
City 15 (vs. City 1)	0.16 (0.10)	[-0.05, 0.35]	-0.04 (0.17)	[-0.39, 0.27]
City 16 (vs. City 1)	-0.04 (0.12)	[-0.26, 0.19]	-0.10 (0.18)	[-0.46, 0.27]
City 17 (vs. City 1)	-0.19 (0.11)	[-0.41, 0.03]	-0.12 (0.17)	[-0.47, 0.21]
City 18 (vs. City 1)	-0.05 (0.10)	[-0.26, 0.15]	-0.03 (0.18)	[-0.36, 0.29]
City 19 (vs. City 1)	0.00 (0.11)	[-0.21, 0.22]	-0.16 (0.17)	[-0.50, 0.16]
City 20 (vs. City 1)	-0.11 (0.11)	[-0.32, 0.10]	-0.09 (0.17)	[-0.43, 0.24]
Intercepts	0.00 (0.01)	[-0.03, 0.03]	-0.01 (0.02)	[-0.05, 0.04]
Random effects				
Variance – outcome	0.27 (0.04)*	[0.21, 0.37]	0.50 (0.16)*	[0.31, 0.86]
Covariance – intercept and slope	0.28 (0.02)*	[0.25, 0.31]	--	--

Note: * *Bayesian significance,* after we used the Benjamini-Hochberg FDR method to account for multiple testing.

© 2024 Del Toro J et al. *JAMA Pediatrics.*

eTable 10

Posterior Estimates (SD) from a Bayesian Multilevel Model Examining Residualized Changes in Epigenetic Age Acceleration among 2,039 Youth Transitioning Between Childhood and Adolescence

	Police intrusion		Age-15 Horvath EAA measure		Age-15 Hannum EAA measure	
Measures	*Estimate (SD)*	*95% CI*	*Estimate (SD)*	*95% CI*	*Estimate (SD)*	*95% CI*
Fixed effects						
Age-9 EAA measure	--	--	1.14 (0.03)*	[1.09, 1.20]	0.62 (0.05)*	[0.53, 0.72]
Police intrusion	--	--	0.02 (0.05)	[-0.08, 0.12]	0.44 (0.16)*	[0.13, 0.76]
Black (vs. White)	0.25 (0.07)*	[0.12, 0.40]	-0.22 (0.16)	[-0.52, 0.09]	0.94 (0.50)	[-0.01, 1.90]
Other (vs. White)	0.04 (0.07)	[-0.11, 0.18]	-0.15 (0.16)	[-0.47, 0.16]	0.48 (0.51)	[-0.49, 1.46]
Latinx (vs. White)	0.03 (0.09)	[-0.14, 0.20]	-0.22 (0.19)	[-0.60, 0.15]	0.81 (0.60)	[-0.35, 1.97]
Female (vs. male)	-0.42 (0.04)*	[-0.50, -0.34]	-0.12 (0.09)	[-0.32, 0.05]	-0.51 (0.29)	[-1.12, 0.04]
Child age	-0.04 (0.06)	[-0.17, 0.08]	-0.30 (0.13)	[-0.56, -0.04]	-0.04 (0.41)	[-0.83, 0.78]
Family SES	-0.13 (0.03)*	[-0.18, -0.08]	-0.05 (0.06)	[-0.17, 0.07]	0.09 (0.18)	[-0.27, 0.46]
Child smoking	0.24 (0.22)	[-0.20, 0.67]	0.60 (0.49)	[-0.37, 1.52]	1.91 (1.52)	[-1.00, 4.83]
Maternal smoking	0.22 (0.05)*	[0.11, 0.33]	0.11 (0.12)	[-0.13, 0.35]	0.13 (0.38)	[-0.61, 0.85]
DNAm smoking	-0.02 (0.02)	[-0.06, 0.02]	0.05 (0.05)	[-0.04, 0.15]	-0.49 (0.15)*	[-0.78, -0.19]
Child BMI	-0.02 (0.02)	[-0.06, 0.02]	0.10 (0.04)	[0.02, 0.18]	0.04 (0.13)	[-0.21, 0.29]
Child puberty	-0.01 (0.04)	[-0.09, 0.06]	-0.03 (0.09)	[-0.19, 0.14]	-0.01 (0.27)	[-0.52, 0.52]
EPIC (vs. 450K)	-0.06 (0.05)	[-0.15, 0.03]	0.02 (0.10)	[-0.18, 0.21]	-0.11 (0.31)	[-0.71, 0.48]
Prop. of epithelial cells	-0.02 (3.43)	[-6.77, 6.77]	-0.47 (7.40)	[-15.59, 13.53]	-6.16 (22.82)	[-53.00, 37.31]
Prop. of fibroblasts	3.46 (4.08)	[-4.63, 11.73]	-0.87 (8.63)	[-18.12, 15.61]	30.85 (26.57)	[-22.29, 81.49]
Prop. of immune cells	-0.22 (3.43)	[-6.94, 6.57]	0.66 (7.40)	[-14.26, 14.68]	6.58 (22.81)	[-39.61, 49.53]
City 2 (vs. City 1)	0.01 (0.12)	[-0.23, 0.25]	-0.02 (0.27)	[-0.57, 0.50]	0.32 (0.83)	[-1.35, 1.96]
City 3 (vs. City 1)	0.03 (0.13)	[-0.22, 0.29]	-0.24 (0.28)	[-0.80, 0.30]	0.15 (0.87)	[-1.60, 1.83]

© 2024 Del Toro J et al. *JAMA Pediatrics.*

City 4 (vs. City 1)	-0.11 (0.12)	[-0.35, 0.14]	-0.43 (0.27)	[-0.94, 0.09]	-0.20 (0.83)	[-1.81, 1.44]
City 5 (vs. City 1)	-0.15 (0.13)	[-0.39, 0.10]	-0.11 (0.28)	[-0.65, 0.44]	0.25 (0.87)	[-1.42, 1.91]
City 6 (vs. City 1)	0.04 (0.13)	[-0.21, 0.30]	-0.15 (0.28)	[-0.69, 0.40]	-0.03 (0.86)	[-1.28, 2.04]
City 7 (vs. City 1)	-0.14 (0.12)	[-0.39, 0.10]	-0.31 (0.27)	[-0.86, 0.22]	0.40 (0.84)	[-1.28, 2.04]
City 8 (vs. City 1)	-0.12 (0.12)	[-0.35, 0.11]	-0.09 (0.26)	[-0.61, 0.44]	-0.02 (0.81)	[-1.63, 1.60]
City 9 (vs. City 1)	-0.16 (0.12)	[-0.42, 0.08]	-0.15 (0.26)	[-0.68, 0.34]	-0.26 (0.81)	[-1.91, 1.27]
City 10 (vs. City 1)	-0.07 (0.12)	[-0.31, 0.16]	-0.57 (0.26)	[-1.08, -0.06]	0.15 (0.81)	[-1.46, 1.71]
City 11 (vs. City 1)	0.02 (0.13)	[-0.24, 0.29]	-0.43 (0.29)	[-1.03, 0.13]	-1.41 (0.91)	[-3.22, 0.34]
City 12 (vs. City 1)	-0.11 (0.12)	[-0.35, 0.13]	0.03 (0.28)	[-0.52, 0.56]	-0.59 (0.86)	[-2.26, 1.07]
City 13 (vs. City 1)	-0.08 (0.17)	[-0.41, 0.25]	-0.23 (0.39)	[-0.99, 0.53]	0.84 (1.20)	[-1.52, 3.19]
City 14 (vs. City 1)	-0.34 (0.16)	[-0.68, -0.02]	-0.46 (0.37)	[-1.20, 0.23]	-0.88 (1.13)	[-3.16, 1.25]
City 15 (vs. City 1)	0.20 (0.16)	[-0.12, 0.52]	-0.50 (0.35)	[-1.20, 0.18]	-0.42 (1.09)	[-2.59, 1.66]
City 16 (vs. City 1)	-0.03 (0.18)	[-0.36, 0.34]	-0.71 (0.39)	[-1.46, 0.08]	-0.74 (1.19)	[-3.10, 1.69]
City 17 (vs. City 1)	0.02 (0.16)	[-0.30, 0.32]	-0.55 (0.35)	[-1.25, 0.15]	-1.09 (1.09)	[-3.24, 1.07]
City 18 (vs. City 1)	-0.11 (0.16)	[-0.42, 0.21]	-0.48 (0.35)	[-1.17, 0.23]	-0.02 (1.08)	[-2.14, 2.15]
City 19 (vs. City 1)	-0.19 (0.16)	[-0.52, 0.13]	-0.23 (0.36)	[-0.97, 0.47]	-0.26 (1.11)	[-2.56, 1.91]
City 20 (vs. City 1)	-0.34 (0.17)	[-0.66, -0.01]	-0.19 (0.36)	[-0.89, 0.52]	-0.39 (1.10)	[-2.55, 1.91]
Intercepts	0.00 (0.02)	[0.00, 0.02]	0.04 (0.05)	[-0.05, 0.14]	-0.04 (0.14)	[-0.31, 0.25]
Random effects						
Variance – outcome	0.81 (0.03)*	[0.76, 0.87]	3.82 (0.12)*	[3.59, 4.07]	36.29 (1.19)*	[34.06, 38.63]

Note: * *Bayesian significance*, after we used the Benjamini-Hochberg FDR method to account for multiple testing.

© 2024 Del Toro J et al. *JAMA Pediatrics.*

eTable 11

Posterior Estimates (SD) from a Bayesian Multilevel Model Examining Residualized Changes in Epigenetic Age Acceleration among 2,039 Youth Transitioning Between Childhood and Adolescence

	Age-15 GrimAge EAA measure		Age-15 PhenoAge EAA measure		Age-15 DunedinPACE EAA measure	
Measures	*Estimate (SD)*	*95% CI*	*Estimate (SD)*	*95% CI*	*Estimate (SD)*	*95% CI*
Fixed effects						
Age-9 EAA measure	0.73 (0.04)*	[0.64, 0.81]	0.59 (0.02)*	[0.54, 0.63]	0.42 (0.04)*	[0.35, 0.49]
Police intrusion	0.36 (0.10)*	[0.17, 0.56]	0.35 (0.12)*	[0.11, 0.59]	0.06 (0.02)*	[0.01, 0.11]
Black (vs. White)	1.28 (0.31)*	[0.68, 1.99]	0.14 (0.38)	[-0.59, 0.89]	0.37 (0.07)*	[0.23, 0.52]
Other (vs. White)	0.81 (0.32)*	[0.18, 1.42]	0.10 (0.40)	[-0.67, 0.85]	0.20 (0.08)*	[0.05, 0.35]
Latinx (vs. White)	0.65 (0.38)	[-0.08, 1.39]	0.39 (0.47)	[-0.51, 1.30]	0.25 (0.09)*	[0.07, 0.42]
Female (vs. male)	0.32 (0.19)	[-0.06, 0.68]	1.23 (0.23)*	[0.75, 1.67]	0.26 (0.04)*	[0.17, 0.34]
Child age	-0.36 (0.26)	[-0.87, 0.15]	-0.01 (0.32)	[-0.63, 0.61]	-0.05 (0.06)	[-0.17, 0.07]
Family SES	-0.05 (0.12)	[-0.28, 0.18]	-0.01 (0.14)	[-0.30, 0.27]	-0.02 (0.03)	[-0.08, 0.03]
Child smoking	0.65 (0.96)	[-1.19, 2.49]	-0.58 (1.18)	[-2.83, 1.69]	0.34 (0.23)	[-0.09, 0.78]
Maternal smoking	0.08 (0.24)	[-0.39, 0.54]	0.20 (0.29)	[-0.37, 0.77]	0.05 (0.06)	[-0.06, 0.16]
DNAm smoking	-0.24 (0.11)	[-0.46, -0.01]	-0.14 (0.12)	[-0.37, 0.10]	-0.07 (0.02)*	[-0.11, -0.03]
Child BMI	0.13 (0.08)	[-0.03, 0.29]	0.22 (0.10)	[0.03, 0.42]	0.07 (0.02)*	[0.03, 0.11]
Child puberty	-0.09 (0.17)	[-0.41, 0.25]	-0.07 (0.21)	[-0.46, 0.34]	-0.02 (0.04)	[-0.10, 0.06]
EPIC (vs. 450K)	-0.19 (0.20)	[-0.56, 0.19]	0.03 (0.24)	[-0.44, 0.49]	-0.06 (0.05)	[-0.15, 0.03]
Prop. of epithelial cells	-4.72 (14.40)	[-34.17, 22.74]	-1.79 (17.79)	[-38.19, 32.01]	-0.61 (3.44)	[-7.67, 5.93]
Prop. of fibroblasts	33.58 (16.79)	[-0.26, 65.50]	33.20 (20.87)	[-9.19, 72.70]	1.98 (4.02)	[-5.95, 9.68]
Prop. of immune cells	4.94 (14.40)	[-24.24, 32.06]	2.08 (17.80)	[-33.94, 35.70]	0.68 (3.44)	[-6.28, 7.17]
City 2 (vs. City 1)	-0.33 (0.52)	[-1.39, 0.69]	0.04 (0.65)	[-1.27, 1.30]	0.01 (0.12)	[-0.25, 0.25]
City 3 (vs. City 1)	0.13 (0.55)	[-0.95, 1.20]	0.24 (0.68)	[-1.11, 1.55]	0.01 (0.13)	[-0.25, 0.27]

© 2024 Del Toro J et al. *JAMA Pediatrics.*

City 4 (vs. City 1)	0.08 (0.53)	[-0.92, 1.11]	0.10 (0.65)	[-1.15, 1.37]	0.04 (0.13)	[-0.20, 0.29]
City 5 (vs. City 1)	0.18 (0.55)	[-0.88, 1.23]	0.38 (0.68)	[-0.93, 1.68]	-0.03 (0.13)	[-0.28, 0.23]
City 6 (vs. City 1)	0.23 (0.54)	[-0.83, 1.31]	-0.11 (0.67)	[-1.43, 1.22]	-0.01 (0.13)	[-0.26, 0.25]
City 7 (vs. City 1)	0.21 (0.53)	[-0.85, 1.25]	0.08 (0.66)	[-1.24, 1.36]	-0.01 (0.13)	[-0.26, 0.24]
City 8 (vs. City 1)	-0.18 (0.51)	[-1.19, 0.84]	0.50 (0.63)	[-0.75, 1.76]	-0.09 (0.12)	[-0.34, 0.15]
City 9 (vs. City 1)	0.03 (0.51)	[-0.99, 1.00]	-0.16 (0.63)	[-1.45, 1.03]	-0.12 (0.12)	[-0.37, 0.11]
City 10 (vs. City 1)	0.49 (0.51)	[-0.51, 1.47]	0.15 (0.63)	[-1.09, 1.37]	0.08 (0.12)	[-0.16, 0.31]
City 11 (vs. City 1)	-0.48 (0.57)	[-1.65, 0.61]	-1.31 (0.71)	[-2.72, 0.05]	-0.28 (0.14)	[-0.56, -0.02]
City 12 (vs. City 1)	-0.07 (0.54)	[-1.12, 0.97]	0.07 (0.67)	[-1.23, 1.36]	-0.16 (0.13)	[-0.42, 0.08]
City 13 (vs. City 1)	-0.16 (0.75)	[-1.66, 1.32]	-0.21 (0.93)	[-2.05, 1.63]	-0.10 (0.18)	[-0.46, 0.26]
City 14 (vs. City 1)	-0.44 (0.71)	[-1.88, 0.90]	-1.18 (0.88)	[-2.96, 0.48]	-0.22 (0.17)	[-0.56, 0.10]
City 15 (vs. City 1)	-0.11 (0.69)	[-1.48, 1.21]	-0.14 (0.85)	[-1.83, 1.48]	0.04 (0.16)	[-0.29, 0.35]
City 16 (vs. City 1)	-0.54 (0.75)	[-1.98, 1.00]	-1.53 (0.93)	[-3.37, 0.36]	-0.11 (0.18)	[-0.46, 0.26]
City 17 (vs. City 1)	-0.63 (0.69)	[-1.99, 0.71]	-0.21 (0.85)	[-1.89, 1.48]	-0.20 (0.16)	[-0.52, 0.12]
City 18 (vs. City 1)	-0.23 (0.68)	[-1.57, 1.14]	0.42 (0.85)	[-1.23, 2.12]	-0.05 (0.16)	[-0.37, 0.28]
City 19 (vs. City 1)	0.10 (0.70)	[-1.34, 1.48]	-0.28 (0.87)	[-2.07, 1.42]	-0.07 (0.17)	[-0.41, 0.26]
City 20 (vs. City 1)	0.16 (0.70)	[-1.21, 1.54]	-0.58 (0.86)	[-2.27, 1.10]	-0.11 (0.17)	[-0.44, 0.21]
Intercepts	-0.03 (0.09)	[-0.20, 0.16]	0.03 (0.11)	[-0.18, 0.26]	-0.01 (0.02)	[-0.05, 0.04]
Random effects						
Variance – outcome	14.56 (0.47)*	[13.57, 15.38]	22.10 (0.72)*	[20.75, 23.52]	0.82 (0.03)*	[0.77, 0.88]

Note: * *Bayesian significance*, after we used the Benjamini-Hochberg FDR method to account for multiple testing.

© 2024 Del Toro J et al. *JAMA Pediatrics.*

eTable 12

Posterior Estimates (SD) from a Bayesian Multilevel Model Examining Main Effects on Age-15 Direct Police Intrusion among 2,039 Youth

	Age-15 police intrusion	
Measures	*Estimate (SD)*	*95% CI*
Fixed effects		
Age-9 Horvath EAA measure	-0.01 (0.01)	[-0.04, 0.01]
Age-9 Hannum EAA measure	-0.01 (0.01)	[-0.02, 0.01]
Age-9 GrimAge EAA measure	0.01 (0.01)	[-0.01, 0.03]
Age-9 PhenoAge EAA measure	0.00 (0.00)	[-0.01, 0.01]
Age-9 DunedinPACE EAA measure	0.00 (0.04)	[-0.07, 0.08]
Black (vs. White)	0.24 (0.07)*	[0.10, 0.39]
Other (vs. White)	0.03 (0.08)	[-0.12, 0.18]
Latinx (vs. White)	0.03 (0.09)	[-0.14, 0.20]
Female (vs. male)	-0.41 (0.05)*	[-0.50, -0.32]
Child age	-0.04 (0.06)	[-0.16, 0.08]
Family SES	-0.13 (0.03)*	[-0.18, -0.07]
Child smoking	0.24 (0.21)	[-0.18, 0.66]
Maternal smoking	0.22 (0.05)*	[0.11, 0.33]
DNAm smoking	-0.03 (0.03)	[-0.09, 0.02]
Child BMI	-0.02 (0.02)	[-0.06, 0.02]
Child puberty	-0.02 (0.04)	[-0.10, 0.06]
EPIC (vs. 450K)	-0.06 (0.05)	[-0.15, 0.03]
Prop. of epithelial cells	0.08 (3.55)	[-7.11, 7.13]
Prop. of fibroblasts	3.61 (4.28)	[-4.90, 11.81]
Prop. of immune cells	0.04 (3.55)	[-7.13, 7.16]
City 2 (vs. City 1)	0.01 (0.12)	[-0.23, 0.24]
City 3 (vs. City 1)	0.02 (0.13)	[-0.23, 0.27]
City 4 (vs. City 1)	-0.11 (0.12)	[-0.35, 0.13]
City 5 (vs. City 1)	-0.15 (0.13)	[-0.41, 0.10]
City 6 (vs. City 1)	0.04 (0.13)	[-0.20, 0.30]
City 7 (vs. City 1)	-0.13 (0.12)	[-0.37, 0.10]
City 8 (vs. City 1)	-0.12 (0.12)	[-0.35, 0.12]
City 9 (vs. City 1)	-0.16 (0.12)	[-0.39, 0.07]
City 10 (vs. City 1)	-0.08 (0.12)	[-0.30, 0.17]

© 2024 Del Toro J et al. *JAMA Pediatrics.*

City 11 (vs. City 1)	0.01 (0.14)	[-0.25, 0.28]
City 12 (vs. City 1)	-0.11 (0.12)	[-0.35, 0.14]
City 13 (vs. City 1)	-0.07 (0.17)	[-0.40, 0.26]
City 14 (vs. City 1)	-0.33 (0.16)	[-0.65, -0.01]
City 15 (vs. City 1)	0.19 (0.16)	[-0.13, 0.51]
City 16 (vs. City 1)	-0.03 (0.18)	[-0.38, 0.31]
City 17 (vs. City 1)	0.02 (0.17)	[-0.30, 0.35]
City 18 (vs. City 1)	-0.09 (0.16)	[-0.42, 0.22]
City 19 (vs. City 1)	-0.18 (0.16)	[-0.51, 0.14]
City 20 (vs. City 1)	-0.33 (0.16)	[-0.65, -0.02]
Intercepts	0.32 (0.02)*	[0.28, 0.36]
Random effects		
Variance – outcome	0.81 (0.03)*	[0.76, 0.87]

Note: * *Bayesian significance*, after we used the Benjamini-Hochberg FDR method to account for multiple testing.

© 2024 Del Toro J et al. *JAMA Pediatrics.*

eTable 13

Zero-Order Bivariate Correlations Between Principal Components (PCs) and the Horvath Epigenetic Age Acceleration Measure at Ages 9 and 15 among 2,039 Youth

	White youth		Black youth		Latinx youth		Other youth	
PCs	Age-9	Age-15	Age-9	Age-15	Age-9	Age-15	Age-9	Age-15
pc1	.06	.06	-.04	.00	-.04	.01	.14	.10
pc2	.02	-.11	.00	.04	.04	.06	-.14	.05
pc3	.04	.14	.00	.01	-.06	-.08	.03	.04
pc4	.02	-.11	.00	.00	.02	.03	-.11	.00
pc5	.09	.07	.06	.11	-.02	-.03	-.14	-.03
pc6	-.07	.06	.00	.00	-.05	.04	.18	.12
pc7	.09	.04	-.02	-.02	-.01	.00	.06	.06
pc8	-.10	-.05	-.03	.00	.02	.01	.17	.08
pc9	-.06	.00	.02	.01	.02	-.02	.06	.12
pc10	-.06	.01	-.03	-.06	.09	.01	.07	.25
pc11	-.03	-.06	.00	.00	.03	.02	.02	-.04
pc12	.06	-.04	-.02	.00	.02	.01	-.15	-.09
pc13	-.04	.00	-.06	-.06	-.01	.01	.27	-.01
pc14	.05	.00	.00	.03	.03	.05	-.08	-.06
pc15	-.02	-.04	-.08	-.07	.04	-.07	-.24	-.12
pc16	.01	.06	.00	.02	.04	.05	.01	-.07
pc17	-.05	-.07	.05	.04	.05	.03	-.24	-.04
pc18	.07	.07	.02	.03	-.02	-.01	-.03	-.03
pc19	.03	-.03	.00	.02	-.01	-.03	.10	.06
pc20	.04	.05	-.01	-.01	-.03	.00	-.08	.00

Note: * $p < .05$, ** $p < .01$, *** $p < .001$, after we used the Benjamini-Hochberg FDR method to account for multiple testing.

© 2024 Del Toro J et al. *JAMA Pediatrics.*

eTable 14

Zero-Order Bivariate Correlations Between Principal Components (PCs) and the Hannum Epigenetic Age Acceleration Measure at Ages 9 and 15 among 2,039 Youth

	White youth		Black youth		Latinx youth		Other youth	
PCs	Age-9	Age-15	Age-9	Age-15	Age-9	Age-15	Age-9	Age-15
pc1	.06	-.05	.01	-.02	-.04	-.06	.18	.14
pc2	-.03	-.08	-.01	.03	.01	-.10	-.17	.07
pc3	-.06	.01	-.01	.03	-.02	.11	-.02	-.17
pc4	-.07	-.07	-.01	.01	-.04	-.02	-.14	-.11
pc5	.04	.02	.04	.03	-.03	-.07	-.10	.12
pc6	.05	.01	.03	-.04	.03	.05	.06	.02
pc7	-.04	-.03	-.01	.03	.04	.02	.01	-.18
pc8	.00	-.08	-.01	.02	-.03	-.07	.08	.09
pc9	-.07	-.03	-.05	-.04	.03	.03	-.13	-.06
pc10	.03	.03	.08	-.03	-.01	-.07	.00	.02
pc11	-.05	-.14	.06	.05	.05	.08	.02	.07
pc12	.18	.07	-.08	.00	.03	-.02	-.11	-.10
pc13	-.01	-.01	.04	.02	-.01	-.02	.18	-.10
pc14	-.04	-.07	-.03	.00	.00	.01	-.10	-.01
pc15	.04	.06	.04	.04	.03	-.03	-.11	-.10
pc16	.06	-.02	.01	.01	-.12	-.03	.05	-.04
pc17	-.09	.00	.05	.06	-.02	-.01	-.20	.18
pc18	.00	-.03	.05	.06	-.05	.06	.15	-.13
pc19	.05	.09	-.03	-.03	-.08	-.02	.05	.12
pc20	.08	.10	.02	.00	-.03	-.01	-.18	.02

Note: * $p < .05$, ** $p < .01$, *** $p < .001$, after we used the Benjamini-Hochberg FDR method to account for multiple testing.

© 2024 Del Toro J et al. *JAMA Pediatrics.*

eTable 15

Zero-Order Bivariate Correlations Between Principal Components (PCs) and the GrimAge Epigenetic Age Acceleration Measure at Ages 9 and 15 among 2,039 Youth

	White youth		Black youth		Latinx youth		Other youth	
PCs	Age-9	Age-15	Age-9	Age-15	Age-9	Age-15	Age-9	Age-15
pc1	.03	-.01	.01	.02	.00	-.01	.07	.27
pc2	-.03	-.04	-.02	.01	-.05	-.16	-.08	-.02
pc3	.00	.04	.01	.03	.03	.16	-.03	-.14
pc4	-.05	-.05	-.02	-.02	.03	.04	-.05	-.04
pc5	.04	.01	.04	.03	.04	.00	-.07	.01
pc6	.03	-.03	.00	-.06	.03	-.01	.00	.11
pc7	-.01	.00	.03	.05	.00	-.01	-.11	-.05
pc8	-.01	-.08	-.01	.04	-.04	-.03	.11	.21
pc9	-.09	-.05	-.01	-.03	.05	.02	-.02	.16
pc10	-.02	-.01	.07	-.01	-.01	.01	.01	-.02
pc11	-.06	-.13	.01	.04	.08	.07	.00	.02
pc12	.16	.10	-.10	.02	.03	-.04	.04	-.18
pc13	.00	-.09	.00	.00	.02	.05	.14	-.07
pc14	.00	-.06	-.02	.00	.00	.01	-.01	-.11
pc15	.05	.10	.02	.02	.04	-.01	-.07	-.24
pc16	.06	.00	.04	.02	-.15	-.02	.19	-.02
pc17	-.07	.07	.03	.05	.00	.03	-.02	.08
pc18	.02	-.02	.04	.02	-.03	-.01	.13	-.24
pc19	-.02	.09	.01	.00	-.08	-.01	-.04	.25
pc20	.06	.03	.01	-.03	.01	.06	.00	-.10

Note: * $p < .05$, ** $p < .01$, *** $p < .001$, after we used the Benjamini-Hochberg FDR method to account for multiple testing.

© 2024 Del Toro J et al. *JAMA Pediatrics.*

eTable 16

Zero-Order Bivariate Correlations Between Principal Components (PCs) and the PhenoAge Epigenetic Age Acceleration Measure at Ages 9 and 15 among 2,039 Youth

	White youth		Black youth		Latinx youth		Other youth	
PCs	Age-9	Age-15	Age-9	Age-15	Age-9	Age-15	Age-9	Age-15
pc1	.09	.06	.01	.02	-.10	-.13	-.14	-.01
pc2	-.02	-.04	-.04	.04	-.06	-.12	.06	-.04
pc3	-.07	.01	-.05	-.03	.04	.11	.07	-.19
pc4	-.04	-.06	-.02	.00	-.01	.02	-.02	-.12
pc5	-.02	.05	.06	.11	.00	-.04	-.15	.20
pc6	.06	.03	.04	-.02	-.02	.00	-.23	-.06
pc7	-.03	-.06	.03	.06	.08	.02	-.04	.07
pc8	-.05	-.03	.02	.04	-.01	-.01	-.15	-.14
pc9	.03	-.02	-.05	-.04	.04	.04	-.22	.11
pc10	-.08	-.03	.02	-.05	.07	.04	-.09	.28
pc11	-.10	-.04	.00	.05	.04	.06	-.03	-.24
pc12	.07	.05	-.09	-.04	.03	.03	.24	.07
pc13	-.01	.01	-.04	-.05	-.04	.01	.18	-.35
pc14	.05	-.07	.01	-.01	-.01	.06	.28	.02
pc15	.00	.04	.00	.02	.10	.03	.11	-.22
pc16	-.01	.01	.02	.04	-.01	.01	.10	-.14
pc17	-.04	-.06	.04	.01	.04	.01	-.15	-.01
pc18	.06	.05	.01	-.01	-.02	-.03	.37	.02
pc19	-.05	.01	-.02	.00	-.03	-.02	-.28	.10
pc20	.05	.06	.00	.00	-.01	-.03	.08	-.02

Note: * $p < .05$, ** $p < .01$, *** $p < .001$, after we used the Benjamini-Hochberg FDR method to account for multiple testing.

eTable 17

Zero-Order Bivariate Correlations Between Principal Components (PCs) and the DunedinPACE Epigenetic Age Acceleration Measure at Ages 9 and 15 among 2,039 Youth

	White youth		Black youth		Latinx youth		Other youth	
PCs	Age-9	Age-15	Age-9	Age-15	Age-9	Age-15	Age-9	Age-15
pc1	.07	-.02	.06	.04	-.09	-.12	.09	.28
pc2	-.02	-.05	-.01	.02	-.09	-.19***	-.03	.16
pc3	-.06	.00	.02	.02	.09	.22***	.14	.10
pc4	-.08	-.07	-.03	.01	.06	.02	.09	.16
pc5	-.01	-.01	.02	.01	.07	.02	-.11	-.15
pc6	.11	.01	.04	-.01	.03	.01	.01	.22
pc7	-.03	-.01	-.01	.03	.00	-.02	-.07	-.09
pc8	.05	-.06	.01	.03	-.01	-.04	.11	.29
pc9	-.10	-.04	-.06	-.05	-.04	.00	-.19	.10
pc10	.00	-.01	.06	-.03	-.02	.00	-.09	-.12
pc11	-.02	-.07	-.02	.00	.01	.01	.17	.23
pc12	.18	.05	-.08	.03	.01	-.03	-.03	-.24
pc13	-.01	-.03	.05	.01	.00	-.03	.11	-.02
pc14	.01	-.08	.00	.02	-.05	.01	-.07	-.08
pc15	.04	.06	.04	.06	.02	.04	.08	-.16
pc16	.04	-.03	.04	.00	-.11	.00	.14	-.03
pc17	.01	.07	.03	.03	.00	-.03	-.09	-.01
pc18	.00	-.07	-.01	.05	.00	.04	.17	-.29
pc19	.06	.15	-.05	-.06	-.04	.02	.03	.24
pc20	.02	.00	.01	.00	.02	.05	-.17	-.16

Note: * $p < .05$, ** $p < .01$, *** $p < .001$, after we used the Benjamini-Hochberg FDR method to account for multiple testing.

© 2024 Del Toro J et al. *JAMA Pediatrics.*
