# Supplementary material for: Longitudinal Changes in Epigenetic Age Acceleration Across Childhood and Adolescence
Source: JAMA Pediatr. 2024 Oct 7;178(12):1298–306. doi: 10.1001/jamapediatrics.2024.3669 (PMC11459359; doi:10.1001/jamapediatrics.2024.3669)
Supplement: Supplement 2. — Data sharing statement [file jamapediatr-e243669-s002.pdf]

Data Sharing Statement

Del Toro. Longitudinal Changes in Epigenetic Age Acceleration Across Childhood and Adolescence. *JAMA Pediatr*. Published October 07, 2024. doi:10.1001/jamapediatrics.2024.3669

Data
Data available: No

Additional Information
Explanation for why data not available: The data are publicly available via the Future of Families and Child Wellbeing Study archive.
